# Guidelines for the standardization of pre‐analytical variables for salivary biomarker studies in Alzheimer's disease research: An updated review and consensus of the Salivary Biomarkers for Dementia Research Working Group

**DOI:** 10.1002/alz.14420

**Published:** 2024-12-30

**Authors:** Ted K. S. Ng, Chinedu Udeh‐Momoh, Mei‐Ann Lim, Helena Sophia Gleerup, Wayne Leifert, Catherine Ajalo, Nicholas Ashton, Henrik Zetterberg, Robert A. Rissman, Charisse N. Winston, Sid O’ Bryant, Robert Jenkins, Eva Carro, Gorka Orive, Stefano Tamburin, Marcos Olvera‐Rojas, Patricio Solis‐Urra, Irene Esteban‐Cornejo, Gustavo Alves Andrade Dos Santos, Kumar B. Rajan, David Koh, Anja Hviid Simonsen, Paul D. Slowey, the Alzheimer's Association International Society to Advance Alzheimer's Research and Treatment: Biofluid Based Biomarkers Professional Interest Area Salivary Biomarkers for Dementia Research Working Group (ISTAART‐BBB‐PIA‐SWG)

**Affiliations:** ^1^ Rush Institute for Healthy Aging Department of Internal Medicine Rush University Medical Center Chicago Illinois USA; ^2^ Edson College of Nursing and Health Education Arizona State University Phoenix Arizona USA; ^3^ School of Public Health Sciences Wake Forest University School of Medicine, North Carolina Medical Center Boulevard Winston‐Salem North Carolina USA; ^4^ Brain and Mind Institute Aga Khan University Nairobi Kenya; ^5^ Division of Clinical Geriatrics Center for Alzheimer Research Karolinska Institutet Stockholm Stockholm Sweden; ^6^ Sheffield Institute for Translational Neuroscience (SITraN) University of Sheffield Sheffield UK; ^7^ PAPRSB Institute of Health Sciences Universiti Brunei Darussalam Darussalam Brunei; ^8^ Danish Dementia Research Centre (DDRC) Department of Neurology Copenhagen University Hospital Rigshospitalet Copenhagen Denmark; ^9^ Molecular Diagnostic Solutions Group Human Health Program Commonwealth Scientific and Industrial Research Organisation (CSIRO) Health and Biosecurity Adelaide South Australia Australia; ^10^ School of Biological Sciences The University of Adelaide Adelaide South Australia Australia; ^11^ Banner Health Foundation and Banner Alzheimer's Foundation Phoenix Arizona USA; ^12^ Department of Psychiatry and Neurochemistry Institute of Neuroscience and Physiology the Sahlgrenska Academy at the University of Gothenburg Mölndal Sweden; ^13^ Clinical Neurochemistry Laboratory Sahlgrenska University Hospital Mölndal Sweden; ^14^ Department of Neurodegenerative Disease UCL Institute of Neurology Queen Square London UK; ^15^ UK Dementia Research Institute at UCL Maple House London UK; ^16^ Hong Kong Center for Neurodegenerative Diseases Clear Water Bay Science Park Hong Kong China; ^17^ Wisconsin Alzheimer's Disease Research Center University of Wisconsin School of Medicine and Public Health University of Wisconsin‐Madison Madison Wisconsin USA; ^18^ Department of Physiology and Neuroscience and Alzheimer's Disease Therapeutic Research Institute Keck School of Medicine of the University of Southern California Los Angeles California USA; ^19^ Family Medicine and Osteopathic Manipulative Medicine Institute for Healthy Aging Institute for Translational Research Texas College of Osteopathic Medicine UNT Health Science Center Fort Worth Texas USA; ^20^ Geroa Diagnostics, Vitoria‐Gasteiz Vitoria‐Gasteiz Spain; ^21^ Neurobiology of Alzheimer's Disease Unit Functional Unit for Research into Chronic Diseases Instituto de Salud Carlos III Madrid Spain; ^22^ Network Centre for Biomedical Research in Neurodegenerative Diseases (CIBERNED) ISCIII Fuencarral‐El Pardo Madrid Spain; ^23^ NanoBioCel Research Group School of Pharmacy University of the Basque Country (UPV/EHU) Vitoria‐Gasteiz Spain; ^24^ Bioaraba, NanoBioCel Research Group Vitoria‐Gasteiz Spain; ^25^ Biomedical Research Networking Centre in Bioengineering Biomaterials and Nanomedicine (CIBER‐BBN) Institute of Health Carlos III Madrid Spain; ^26^ University Institute for Regenerative Medicine and Oral Implantology‐UIRMI (UPV/EHU‐Fundación Eduardo Anitua) Vitoria‐Gasteiz Spain; ^27^ Department of Neurosciences Biomedicine, and Movement Sciences University of Verona Verona Italy; ^28^ Department of Physical Education and Sports Faculty of Sport Sciences Sport and Health University Research Institute (iMUDS) University of Granada Granada Spain; ^29^ Faculty of Education and Social Sciences University of Andres Bello Viña del Mar Chile; ^30^ CIBER de Fisiopatología de la Obesidad y Nutrición (CIBEROBN) Instituto de Salud Carlos III Granada Spain; ^31^ Instituto de Investigación Biosanitaria ibs.GRANADA Beiro Granada Spain; ^32^ University of Sao Paulo and Sao Leopoldo Mandic School of Medicine São Paulo São Paulo Brazil; ^33^ Saw Swee Hock School of Public Health National University of Singapore Singapore Singapore; ^34^ Oasis Diagnostics® Corporation Vancouver Washington USA; ^35^ Central South University Changsha Hunan China; ^36^ RapidDx, Inc. Milwaukee New Berlin Wisconsin USA

**Keywords:** Alzheimer's disease, biomarkers, measurement protocol, non‐invasive, pre‐analytical variables, saliva, sampling

## Abstract

There is a pressing need for accessible biomarkers with high diagnostic accuracy for Alzheimer's disease (AD) diagnosis to facilitate widespread screening, particularly in underserved groups. Saliva is an emerging specimen for measuring AD biomarkers, with distinct contexts of use that could complement blood and cerebrospinal fluid and detect various analytes. An interdisciplinary, international group of AD and related dementias (ADRD) researchers convened and performed a narrative review of published studies on salivary AD biomarkers. We critically appraised the current state of the literature, examining both consistencies and discrepancies in existing pre‐analytical variables and methodologies. We discussed how various pre‐analytical variables could influence the detection and quantification of salivary biomarkers, showed technologies available to standardize collection procedures, and proposed a standardized pre‐analytical protocol to guide future studies on salivary AD biomarker examinations. We identified potential contexts of use, gaps, and priorities and proposed future research directions.

**Highlights:**

- Given its non‐invasive nature, wider accessibility, and cultural acceptability, particularly in low‐resourced settings, saliva is a biofluid complementary to blood and CSF.
- Current salivary AD biomarker studies do not control for many confounding pre‐analytical variables during the sampling process, potentially leading to inaccurate salivary biomarker readings and conclusions, contributing to conflicting findings.
- Reviewing the current literature, including the consistencies and non‐consistencies observed in the existing parameters and methodologies, discussing how they can affect salivary AD biomarker detection and quantification.
- Proposing a standardized salivary pre‐analytical protocol, identifying the gaps and prioritizations needed to move this area forward, proposing future directions and potential contexts of use.

## 1 BACKGROUND

The prevailing hypothesis of Alzheimer's disease (AD) is that pathogenic amyloid beta (Aβ) acts as an initiating factor triggering the accumulation of downstream neuropathologies, including tau, and consequent neurodegeneration and dementia.^1^, ^2^, ^3^ Worldwide population aging, resulting in an increased incidence of AD, is prompting the need for faster, cheaper, and more decentralized testing approaches, including inpatient, outpatient clinic, and in‐home testing.

Peripheral biomarkers of AD and neurodegeneration with high diagnostic and prognostic utility are crucial for a cost‐effective, high‐throughput, and translatable paradigm and are needed in population‐based studies, clinical care, and clinical trials, especially in determining the choice of therapeutic targets, development of drug candidates, and design of efficient clinical trials, including participant selection. However, assessments of Aβ and pathological tau accumulation in the brain using positron emission tomography (PET) scan, the gold standard for *ante mortem* detection of AD pathology,^4^ are expensive and not easily accessible, particularly in rural areas and underdeveloped countries, whereas cerebrospinal fluid (CSF) sampling suffers the same drawbacks of PET scan and in addition faces resistance from patients and research participants due to its invasive nature and cultural considerations.

The AT(N) system was proposed to stage AD neuropathology through amyloid, tau, and neurodegeneration in vivo biomarkers. This biomarker matrix has been recently expanded into an ATX(N) system, where X represents novel candidate biomarkers for additional pathophysiological mechanisms, such as neuroimmune dysregulation, synaptic dysfunction, and blood–brain barrier alterations.^5^ These new biomarkers might not be adequately assessed with PET and neuroimaging, and their inclusion in the AD biomarker portfolio underscores the importance of the availability of less‐invasive and more widely applicable matrixes, like saliva.

Recent advancements in AD biomarkers in biofluids, such as blood, have led to the development of ultra‐sensitive assays with high accuracy for detecting the presence of brain AD pathologies.^6^ While collecting blood samples is indisputably less invasive than collecting CSF via a spinal tap, it presents logistical challenges related to accessibility, such as difficulties with at‐home testing, sample stabilization, transportation, and long‐term serial monitoring. Blood collection and transportation to a laboratory can also be resource‐intensive and logistically challenging in low‐resource and/or rural settings.

Saliva, a complementary biofluid, holds promise given its non‐invasive nature and wider accessibility since saliva can be collected independently, remotely, and without nurses and/or specialist‐supported training.^7^, ^8^ Approximately 20% to 30% of proteins found in blood are present in saliva,^9^ with whole saliva containing >2000 proteins, making saliva a medium with enormous potential as a non‐invasive biofluid for the examination of peripheral AD biomarkers.^10^, ^11^ Compared to blood and CSF, saliva sampling is the least invasive, most cost‐effective, simplest method and has more widespread cultural acceptability in specific populations, such as East Asian and African countries.^12^, ^13^, ^14^, ^15^, ^16^ All these advantages could potentially and significantly increase subject enrollment and retention in population and cohort studies and clinical trials, reducing the issue of loss to follow‐up. Saliva is also a universal specimen type that can be used to detect multiple analyte types, including proteins, deoxyribonucleic acid (DNA), ribonucleic acid (RNA), cell‐free components like extracellular vesicles and microRNAs (miRNAs), hormones, metabolites, and others.^15^, ^16^ However, despite all these advantages, saliva sampling is not without its shortcomings. One prominent drawback is the self‐sampling procedure, potentially imposing many confounding variables during the sampling process that could skew the eventual biomarker readings. Other common disadvantages of saliva sampling include the possibility of diurnal variation and inconsistent sampling timing, possible blood contamination, and the potential confounding effects of poor oral health,^15^ while nutritional status or dietary intake seems to have limited effects on saliva testing in non‐AD biomarkers^17^, ^18^; however, their effects on salivary AD biomarkers warrant further investigation. The various collection protocols employed by different laboratories and the lack of standardization in pre‐analytical and post‐analytical parameters further impede cross‐comparisons across laboratories and studies. Since many analytes of interest in AD are protein‐based, stabilization at the collection point is also necessary to ensure sample integrity. These issues have given rise to the urgent need for a standardized protocol. Given further standardizations and additional studies, saliva‐based prediction of brain AD pathology would be especially useful for the identification of at‐risk individuals, have potential application for screening and monitoring purposes in AD trials,^7^ be useful in large‐scale population‐based studies for risk stratifications to facilitate a more targeted prevention approach, and eventually fulfill future diagnostic purposes following full regulatory approval.

A critical step toward generating comparable salivary AD biomarker data across laboratories and studies is establishing guidelines and a standardized protocol for pre‐analytical variables, mirroring the fruitful initiatives already established for CSF and blood AD biomarkers.^19^, ^20^, ^21^, ^22^ Standardization efforts with respect to salivary biomarkers in other fields are ongoing and comparatively well established,^23^, ^24^ and although salivary biomarkers have only recently garnered interest in the AD research communities,^25^ a standardized protocol for pre‐analytical variables for salivary AD biomarker research is important. For example, inconsistent outcomes in the measurements of Aβ42 and Aβ40 have been obtained depending on the sampling methodologies. When collecting saliva using the Salivette collection kit, salivary Aβ42 and Aβ40 were not detected.^26^, ^27^ However, when analyzing unstimulated saliva collected by passive drooling, levels of these peptides were detectable.^28^, ^29^

In this paper, we narratively review the current literature on salivary biomarkers for the detection of AD, including the consistencies and inconsistencies observed in the existing parameters and methodologies, discussing how they can affect salivary biomarker detection and quantification, followed by a proposal for a standardized salivary pre‐analytical protocol to serve as a guideline for future salivary AD biomarker studies. Finally, we conclude this white paper by identifying the gaps and prioritizations needed to move this area forward, suggesting future directions and potential contexts of use.

**RESEARCH IN CONTEXT**

1. **Systematic review**: The authors searched a health science database (PubMed) in conducting literature reviews, summarizing seminal papers examining salivary AD biomarkers.
2. **Interpretations**: While some promising signals and utilities have been reported, current salivary AD biomarker studies did not control for many confounding pre‐analytical variables during the sampling process, potentially leading to inaccurate salivary biomarker readings and conclusions, contributing to conflicting findings.
3. **Future directions**: We narratively reviewed the current literature on salivary biomarkers for the detection of AD, including the consistencies and inconsistencies observed in the existing parameters and methodologies, discussing how they can affect salivary biomarker detection and quantification, followed by a proposal for a standardized salivary pre‐analytical protocol to serve as a guideline for future salivary AD biomarker studies. Lastly, we conclude this white paper by identifying the gaps and prioritizations needed to move this area forward, suggesting future directions and potential contexts of use.

## 2 STATE OF SALIVARY BIOMARKERS FOR ALZHEIMER'S DISEASE RESEARCH APPLICATIONS

### 2.1 Salivary biomarkers of non‐canonical AD hallmarks and pathologies

#### 2.1.1 Overview

Several potential saliva‐based biomarkers of the AD pathological process have been described, with the canonical hallmarks of Aβ and tau and associated markers of axonal injury and brain inflammatory processes,^30^ indicated by neurofilament light chain (NfL) and glial fibrillary acidic protein (GFAP), respectively (reviewed elsewhere^31^, ^32^, ^33^). Other studies have evaluated acetylcholinesterase (AChE) activity, lactoferrin, and other biomarkers.^34^ A review of our literature synthesis on investigations of canonical AD biomarkers in saliva^35^ is presented in Table 1.

**TABLE 1:** Summary of studies examining salivary AD biomarkers and relevant pre‐analytical variables.

	Before collection				During collection			After collection				Analysis and results	
**References**	*Demographics*	*Time of saliva collection*	*Eating/drinking/smoking/brushing of teeth/dental work*	*Physical/psychological stressors*	*Collection method and tubes*	*Temperature during collection*	*Flow rate*	*Addition of protease inhibitor (PI)*	*Visual inspection of sample for contaminants (or by laboratory tests)*	*Temperature/length samples remain in collection tube*	*Centrifugation before storage/storage temperature*	*Assay*	*Results biomarkers*
**Lee et al**.^36^	AD + pre‐AD: *n* = 10 PD: *n* = 1 HC: *n* = 26	ND	ND	ND	ND	ND	ND	ND	ND	ND	ND	ELISA	↑ **Aβ42**, AD/HC *p* < .001
**McGeer et al**.^37^	AD: *n* = 23 Low controls: *n* = 25 High controls (risk for AD): *n* = 6	ND	ND	ND	ND	ND	ND	ND	ND	ND	ND	ELISA	↑ **Aβ42** (AD > high controls > low controls), AD*/*low controls*/*high controls, *p* < .001
**Kim et al**.^28^	AD: *n* = 28 HC: *n* = 17	ND	Participants were asked to rinse their mouth with purified water	ND	Passive drool. Unstimulated saliva (2 to 3 mL) was collected in sterile centrifuge containers, then 2% sodium azide solution was added	ND	ND	ND	ND	ND	1500 rpm for 7 min, stored at −80°C	Immunoassay with nanobeads	↑ **Aβ42**: statistically significant ↑ **Aβ40**: not statistically significant
**Sayer et al**.^38^	AD responders: *n* = 22 AD non‐responders: *n* = 14 HC: *n* = 11	ND	Participants rinsed their mouth with 100 mL still mineral water 5 min prior to sampling	ND	1 mL into sterile plastic container	ND	ND	ND	ND	ND	15,000 rpm for 3 min and stored at −20°C or stored on ice at 4°C before immediate use	Ellman's colorimetric method	↓**AchE activity**, non‐responders*/*HC *p* < .005
**Bakhtiari** ^39^	AD: *n* = 15 HC: *n* = 15	Between 9 a.m. and 12 p.m.	Refrain from eating, drinking, or smoking 1 h prior to sampling. Subjects were asked to rinse their mouth with water 5 min prior to sampling	Collected in a relaxing position	Whole unstimulated saliva was collected by spitting into a 15‐mL falcon tube.	ND	ND	ND	ND	Placed on ice.	Stored at −70°C.	Ellman's colorimetric method	↑ **AchE activity**, AD/HC, *p* = .25
**González‐Sánchez et al**.^40^	Cohort 1: AD: *n* = 25 FTD: *n* = 18 MCI: *n* = 21 HC: *n* = 52 Cohort 2: MCI: *n* = 68 HC: *n* = 74	Samples were collected at a consistent time of day	ND	ND	Unstimulated whole saliva collected in sterile plastic containers precoated with a 2% sodium azide solution.	ND	ND	Samples were treated with protease inhibitor cocktail (Roche) before storage	ND	Placed on ice	600 × *g* for 10 min at 4°C, and stored in 0.5‐mL aliquots at −80°C	Lactoferrin human ELISA kit by Abcam	Cohort 1: **Lactoferrin** in prodromal AD and AD*/*HC AUC: 0.95 **Lactoferrin** in prodromal AD and AD*/*FTD AUC: 0.97 Cohort 2: **Lactoferrin** in prodromal AD*/*HC AUC: 0.93 Sensitivity prodromal AD and AD*/*FTD: 87% Specificity prodromal AD and AD*/*FTD: 91%
**Boston et al**.^41^	AD: *n* = 15 VaD: *n* = 13 HC: *n* = 13	NS	Participants rinsed their mouth with 100 mL still mineral water	ND	1 mL into a sterile plastic container	ND	ND	ND	ND	ND	15,000 rpm for 3 min and stored at −20°C or stored on ice at 4°C before immediate use	Ellman's colorimetric method	No significant difference in AchE between groups
**Pekeles et al**.^42^	Round 1: AD: *n* = 46¨ aMCI: *n* = 55 HC: *n* = 47 Round 2: AD: *n* = 41 FTD: *n* = 16 Neurological patients (other than dementia): *n* = 12 HC (older): *n* = 44 HC (younger): *n* = 76	In the morning	ND	ND	Unstimulated saliva was collected by having the subject spit a sample of 4 to 5 mL into a 50‐mL polypropylene tube	ND	ND	Saliva samples were transferred to another tube with an inhibitor cocktail already in it	ND	Placed on ice with inhibitor cocktail and then transferred to Eppendorf tubes and put in a hot water bath (80°C) for 20 min	5000 or 10,000 rpm for 10 min at 4°C and stored in 0.5‐mL aliquots at −80°C	Western blot	Round 1: ↑ **P‐tau396/t‐tau** at phosphorylation sites S396, S404, and the combination of S400, T403, and T404, *p* < .05 Round 2: ↑ median **p‐tau/396t‐tau** at phosphorylation site S396, *p* value AD*/*HC (older) < .05 Sensitivity S396: 73% Specificity S396: 50% Sensitivity S404: 83% Specificity S404: 30%
**Yilmaz et al**.^43^	AD: *n* = 9 aMCI: *n* = 8 HC: *n* = 12	ND	Refrain from eating, drinking, smoking or using any oral hygiene products at least 1 h prior to collection. Subjects were asked to rinse their mouth with water for 5 min.	ND	Saliva was collected by spitting into 50‐cc falcon tubes.	ND	ND	ND	ND	ND	2600 × *g* for 35 min at 4°C, and stored at −80°C	Proton NMR spectroscopy	↑ **Propionate**, AD/aMCI/HC *p* = 0.034. Regression model for **propionate and acetone** AD/HC: AUC: 0.871 Sensitivity: 90.9% Specificity: 84.2%
**Carro et al**.^44^	Cross‐sectional study: AD: *n* = 80 aMCI: *n* = 44 PD: *m* = 59 HC: *n* = 91 Validation: AD: *n* = 36 MCI: *n* = 15 HC: *n* = 40	ND	ND	ND	Unstimulated whole saliva collected in sterile plastic containers precoated with 2% sodium azide solution	ND	ND	Samples were treated with protease inhibitor cocktail (Roche) before storage	ND	Placed on ice	600 × *g* for 10 min at 4°C, stored in 0.5‐mL aliquots at −80°C	Lactoferrin human ELISA kit by Abcam	Cross‐sectional study: **Lactoferrin** AD/HC *p* < .001, **lactoferrin** aMCI/HC *p* < .001 Validation: Sensitivity: 100%, specificity: 100%
**Peña‐Bautista et al**.^45^	AD: *n* = 14 MCI: *n* = 17 HC: *n* = 12	Collection between 10 a.m. and 12 p.m.	Saliva collection was minimum 30 min after breakfast	ND	Whole saliva was collected by spitting into sterile bottles	ND	ND	ND	Samples with visible blood contamination were excluded from study	ND	Samples were aliquoted into 2‐mL tubes and stored at −80°C	UPLC‐MS/MS	↓ **myo‐inositol and creatine**, *p* = .018 ↑ acetylcholine, *p* = .015
**Gleerup et al**.^46^	AD: *n* = 71 Non‐AD: *n* = 75 MCI: *n* = 56 HC: *n* = 20	Samples were collected from two clinics. One clinic's saliva was collected at noon, while the other's was collected between 9:15 and 10:15 a.m.	Subjects were asked to refrain from drinking, eating, smoking, etc. for at least 30 min prior to sampling. Participants were asked to rinse their mouth with water prior to sampling	ND	Whole unstimulated saliva (1 to 3 mL) was collected in a 15‐mL polypropylene falcon tube	ND	ND	ND	ND	Placed on ice	2000 rpm for 10 min at 4°C, stored in 250‐µL aliquots at −80°C	Lactoferrin human ELISA kit by Abcam and Pierce BCA protein assay kit (Thermo Fisher Scientific)	No significant difference of **lactoferrin, total protein, or normalized lactoferrin** (to total protein) between any groups
**Huan et al**.^47^	Discovery study: AD: *n* = 22 aMCI: *n* = 25 Validation: AD: *n* = 7 aMCI: *n* = 10 HC: *n* = 10	ND	No food in previous hour and light washing prior to saliva collection was administered	ND	Whole saliva was collected and placed inside kit and shaken (Oragene DNA self‐collection kit OG‐500 [DNA Genotek, Canada])	ND	ND	ND	ND	Stored at room temperature	Stored at −80°C	LC‐MS	**Methylguanosine, histidyl‐phenylalanine,** **choline‐cytidine** AD/healthy controls: AUC (discovery and validation) = 1.00 Sensitivity: 100% Specificity: 100% *p* < .01 **Amino‐dihydroxybenzene,** **glucosyl‐galactosyl‐hydroxylysine‐H2O,** **aminobytyric acid + H2** AD/aMCI: AUC (discovery and validation) = 1.00 Sensitivity: 100% Specificity: 100% *p* < .01 **Phenylalanyl‐proline,** **phenylalanyl‐phenylalanine, urocanic acid** AD/healthy controls: AUC discovery: 0.820 AUC validation: 0.814 Sensitivity: 71.4% Specificity: 90.0% *p* < .01 **Alanyl‐phenylalanine, phenylalanyl‐proline** AD/aMCI: AUC discovery: 0.881 AUC validation: 0.786 Sensitivity: 71.4% Specificity: 80.0% *p* < .01
**Liang et al**.^48^	AD: *n* = 256 HC: *n* = 218	Saliva was collected between 9 and 11 a.m.	Subjects were asked to refrain from smoking, eating, and oral hygiene for at least 2 h prior to sampling; subjects were asked to rinse their mouth with water	ND	ND	ND	ND	ND	ND	ND	10,000 rpm for 20 min at 4°C and stored in 200‐µL aliquots at −80°C	FUPLC‐MS	↑ **Spinganine‐1‐phosphate**, *p* < .01 Sensitivity: 99.4% Specificity: 98.2% ↑ **Ornithine**, *p* < .01 Sensitivity: 81.9%
**Ashton et al**.^49^	AD: *n* = 53 aMCI: *n* = 68 HC: *n* = 160	ND	Overnight fasting. Participants rinsed their mouth thoroughly 10 min before saliva collection	ND	Unstimulated saliva was collected into sterile plastic 30‐mL container at 30‐s intervals for 2 min/until 2 mL saliva was collected	ND	ND	ND	ND	ND	500 *g* 10 min at 4°C and stored in 100‐µL aliquots at −80°C	SIMOA	↑ **t‐tau**, although not significant, AD/aMCI/HC *p* = .219
**Lau et al**.^50^	AD: *n* = 20 PD: *n* = 20 HC: *n* = 20	ND	ND	ND	Unstimulated saliva	ND	ND	Protease inhibitor cocktail (Promega Corp., Madison, WI, USA) was added to each supernatant	ND	ND	1000 × *g* for 15 min and stored at −80°C	Trehalose: EG‐IDFET biosensor Aβ42, t‐tau. P‐tau181: ELISA	**Trehalos**e: AD > PD > HC Aβ42: not detected **t‐tau**: no significant differences ↑ **p‐tau181**
**Rai and Kaur. (patent)** ^51^	AD: *n* = 100 HC: *n* = 19	Between 9 and 10 a.m.	Refrain from eating at least 2 h prior to sampling Participants rinsed their mouth with water 10 min prior to sampling	Asked to relax for 5–15 min (during collection)	Unstimulated: passive drool Stimulated: Salivette polyester roll device (Sarstedt, Germany)	ND	Yes, but no significant difference between AD and HC	ND	ND	ND	Unstimulated: 1800 rpm for 5 min Stimulated: 233 rcf for 2 min Both unstimulated and stimulated samples were stored at −80°C	ELISA	↑ **TNF‐α, IL‐1‐β, Aβ42**, *p* < .0001 ↑ **alpha amylase**, *p* < .005 ↓ **Aβ40, IGF‐I, IGF‐II**, *p* < .0001
**Bermejo‐Pareja** ^52^	AD: *n* = 2 PD: *n* = 51 HC (non‐demented without neurological disease or cognitive impairment): *n* = 56	Initiated approximately the same time for each participant (1 p.m.)	Wait at least 4 h after eating or drinking	ND	Collected in sterile plastic containers precoated with 2% sodium azide solution	ND	ND	ND	ND	ND	1500 rpm for 5 min and stored at −80°C	ELISA	**Aβ42**: ↑ concentration in mild (*p* = .043) and moderate AD. AD/HC *p* < .05. Sensitivity: 16% Specificity: 93% **Aβ40**: ↑ concentration in AD and PD
**Gleerup et al**.^53^	AD: *n* = 49 Non‐AD: *n* = 56 MCI: *n* = 47 HC: *n* = 17	Samples were collected from two clinics; one clinic's was collected at noon, the other's between 9:15 and 10:15 a.m.	Subjects were asked to refrain from drinking, eating, smoking, etc. for at least 30 min prior to sampling. Participants were asked to rinse their mouth with water prior to sampling	ND	Whole unstimulated saliva (1 to 3 mL) was collected in 15‐mL polypropylene falcon tube	ND	ND	ND	ND	Placed on ice	2000 rpm for 10 min at 4°C and stored in 250‐µL aliquots at −80°C	SIMOA and Pierce BCA protein assay kit (Thermo Fisher Scientific)	No significant difference of **NfL, total protein or normalized NfL** (to total protein) between any groups
**François et al**.^54^	AD: *n* = 20 MCI: *n* = 20 HC: *n* = 40	ND	ND	ND	Saliva was collected using RNAPro•SAL (Oasis Diagnostics)	ND	ND	ND	ND	ND	Stored at −80°C	Mass spectrometry	Significant changes in **various metabolites and protein from multiple cellular pathways**
**Sabbagh et al**.^29^	AD: *n* = 15 HC: *n* = 7	ND	ND	ND	Passive drool, unstimulated	ND	ND	ND	ND	Stored at room temperature	ND	ELISA	↑ **Aβ42**, *p* < .05
**Shi et al**.^26^	AD: *n* = 21 HC: *n* = 38	ND	Refrained from eating 60 min prior to sampling. Participants rinsed their mouth with water 5 min prior to sampling	Collected in resting state.	Whole saliva. Dental cotton roll was placed between cheek and gum for minimum of 1 min before it was spun in a Salivette (Sarstedt, Germany)	ND	ND	ND	ND	Placed on ice	Stored at −70°C	ELISA (Luminex assay)	**Aβ42**: not detected ↑ **t‐tau** ↑ **p‐tau181** ↑ **p‐tau181/t‐tau** (*p* < .05)
**Katsipis et al**.^30^	AD: *n* = 20 MCI: *n* = 20 HC: *n* = 20	In morning hours	Subjects washed their mouth thoroughly with water	ND	Passive drool, unstimulated	ND	ND	Protease inhibitor cocktail (P‐8849, Sigma‐Aldrich)	ND	ND	13,500 rpm for 15 min; supernatants were vacuum dried overnight and resuspended in 0.01 M phosphate‐buffered saline; samples were stored at −80°C	ELISA and Dot Blot	**GFAP** in AD < HC (*p* < .0001), MCI < HC (*p* < .0001), AD < MCI (Dot Blot; *p* < .001, ELISA; *p* < .0001)
**Zalewska et al**.^55^	AD: *n* = 25 HC: *n* = 25	ND	Subjects were asked to drink glass of water prior to collection	Collected bedside	Whole saliva collected with pipette. The collection was stimulated with 100 µL citric acid sprayed on tip of tongue every 30 s for 10 min	ND	ND	(Addition of 5 µL 0.5 M butylated hydroxytoluene per 0.5 mL salivary supernatant was added to prevent oxidation).	ND	Collected saliva was set aside in a container with ice and was centrifuges within 30 min.	5000 × *g*, 20 min at 4°C and stored at −84°C	Spectrophotometric methods (erythrocyte superoxide dismutase, catalase). Colorimetric method (glutathione)	↓ Activity of **erythrocyte superoxide dismutase, catalase, glutathione peroxidase and glutatione** ↓ Saliva secretion, total protein content ↑ **Aβ**
**Contini et al**.^56^	AD: *n* = 35 Over‐70 HC: *n* = 34 Under‐70 HC: *n* =	ND	ND	ND	ND	ND	ND	ND	ND	ND	ND	LC‐MS	↑ **Alpha‐defensins, thymosin beta4, custatinB, S100A8, A9 in** AD (but all protein/peptides increased with age)
**Sabaei et al**.^57^	Mild AD: 24 First and second stages PD: 24 HC: 22	ND	Subjects had to follow a low‐protein diet and consume fluids frequently 5 days before sampling. Subjects were asked to fast 4 h prior to sampling	ND	Dental cotton roll was placed in participant's mouth	ND	ND	ND	ND	ND	1500 rpm for 5 min and stored at −80°C	ELISA	↑ **Aβ** in PD (*p* < .01) and AD (*p* < .001) ↓**α‐syn** in PD and AD (*p* < .05) ↑ **p‐tau181 i**n AD (*p* < .01)
**Marksteiner et al**.^27^	AD: *n* = 44 MCI: *n* = 45 Depression: *n* = 31 Blinded samples: *n* = 21 HC: *n* = 27	Early in the morning	Subjects were asked to refrain from eating, drinking, smoking, or performing oral hygiene for at least 8 h prior to sampling	ND	Salivette	ND	ND	ND	ND	At room temperature, maximum 4 h	3000 × *g* at room temperature for 5 min and stored at −80°C	Lumipulse	**Aβ40**: not detected **Aβ42**: not detected ↓**Total tau** in AD ↑ **p‐tau181** in MCI
**Ryu et al**.^58^	AD: *n* = 27 HC: *n* = 13	ND	ND	ND	Oral swabs	ND	ND	Swab was placed in tube containing 1 mL phosphate‐buffered saline	ND	ND	12,000 × *g* for 10 min at 4° and stored at −70°C	exoRNeasy Midi Kit	↑ **Salivary EE‐EV**, *p* < .0001 ↑ **miRNA‐485‐3p** in Aβ‐PET‐positive participants (*p* < .0001) and Aβ‐PET‐positive AD patients (*p* = .0063)

Abbreviations: AD, Alzheimer's disease, AUC, area under the curve; AchE, acetylcholinesterase; EG‐IDFET biosensor, extended gate ion‐sensitive field‐effect transistor biosensor; ELISA, enzyme‐linked immunosorbent assay; FTD, frontotemporal dementia; FUPLC‐MS, fast ultraperformance liquid chromatography mass spectrometry; aMCI, amnestic mild cognitive impairment; HC, healthy control; LC‐MS, liquid chromatography mass spectrometry; MCI, mild cognitive impairment; PD, Parkinson disease; SIMOA, single‐molecule array; UPLC‐MS/MS, ultra‐performance liquid chromatography‐tandem mass spectrometry; VaD, vascular dementia; Aβ42, amyloid beta 1‐42; Aβ40, amyloid beta 1‐40; p‐tau, phosphorylated tau; t‐tau, total tau; ↑, increased levels of the biomarker in patients with AD; ↓, decreased levels of the biomarker in patients with AD; ND: not detected.

To produce this review, original studies were identified through a literature search in PubMed for all relevant articles until June 1, 2024. The filters “English” and “humans” were applied, and the following keywords were used for the search: (Saliva) AND diagnos* AND (Alzheimer OR AD) AND (biomarker). To be included, studies must have included a minimum of 10 patients with AD as well as a control group. This resulted in a total of 28 papers^26^, ^27^, ^28^, ^29^, ^30^, ^36^, ^37^, ^38^, ^39^, ^40^, ^41^, ^42^, ^43^, ^44^, ^45^, ^46^, ^47^, ^48^, ^49^, ^50^, ^51^, ^52^, ^53^, ^54^, ^55^, ^56^, ^57^, ^58^ (Table 1).

#### 2.1.2 Salivary Aβ42

In one of the first studies examining salivary AD biomarkers, Sabbagh et al.^29^ quantified salivary Aβ42 levels using an in‐house ELISA assay and found the levels of salivary Aβ42 to be significantly higher than those in cognitively healthy individuals. In 2017, McGeer and co‐workers^36^ described a method for diagnosing AD based on salivary Aβ42 protein and reported levels of Aβ42 in saliva specimens to be double in AD patients compared with normal healthy individuals. They also authored one additional study,^37^ in which salivary Aβ42 levels were quantified in AD patients and normal healthy controls.

Other groups have reported similar findings. For example, work by Rai and Kaur evaluating a series of 50 biomarkers for AD in saliva specimens is the subject of US Patent 9,529,002 from 2016.^37^ The inventors claim specific ratios of the levels of Aβ42 to Aβ40 in saliva specimens as an accurate diagnostic and monitoring tool for AD. Once again, data in this patent support increased levels of Aβ42 and Aβ40 compared to normal healthy individuals and suggest that these biomarkers can be used for diagnostic^21^ and prognostic purposes in cases of mild, moderate, and severe AD forms. This patent includes data on up to 50 biomarkers present in the saliva of AD patients that are detectable and quantifiable in saliva specimens.

Supporting evidence has recently been provided from studies in lower‐resource settings where saliva testing was shown to be beneficial for improving access to diagnosis. Examination of Aβ42 in saliva revealed statistically significant higher levels of Aβ42 in patients with AD,^25^, ^35^ alongside high diagnostic accuracy for discriminating AD patients from controls (area under the curve [AUC] = 0.81),^57^ with sex, but not apolipoprotein E (*APOE)* or age‐related, differences noted in a previous report.^52^ Indeed, saliva Aβ42 levels have been shown to reflect brain AD pathological biomarker levels.^52^ On the other hand, no statistically significant differences between AD patients and controls with salivary Aβ42 have been reported.^52^, ^59^ Inconsistent outcomes in the measurements of Aβ42 and Aβ40 have been found depending on the sampling methodology. When collecting saliva using the Salivette collection kit, salivary Aβ42 and Aβ40 were not detected.^26^, ^27^ However, when analyzing unstimulated saliva collected by passive drooling, levels of these peptides were detectable.^28^, ^29^

Validation of two ELISAs for Aβ42 from commercial suppliers has been performed. The two assays (IBL International, Hamburg, Germany; Biomatik, Kitchener, Canada) are typically provided as kits for serum samples. However, researchers at Oasis Diagnostics Corporation (Vancouver, WA, USA) were able to validate these kits for saliva specimens collected using the Super•SAL Saliva Collection Kit that provides a purified whole saliva specimen with simultaneous removal of mucinous material that can cause downstream assay interference (unpublished results). One of these two assays (IBL International) was used in a randomized controlled trial on mindfulness in mild cognitive impairment (MCI) by Ng et al.^60^, ^61^

Despite these developments, further work on validating salivary AD biomarkers, particularly the salivary Aβ42/40 ratio, is required to explore the diagnostic potential more fully. The authors suggested the crucial need for larger AD cohorts to investigate the reported negative associations and further studies to confirm the sensitivity and specificity of salivary Aβ42 as an AD biomarker.

#### 2.1.3 Salivary total (t‐)tau and phosphorylated (p)Tau181

There have been emerging studies on the diagnostic accuracy of salivary tau biomarkers for detecting AD.^27^, ^57^ Comparing salivary tau biomarker levels in AD patients with those in healthy controls, several studies have reported no significant differences.^49^, ^62^ In contrast, Marksteiner et al. in 2002 reported that, compared to cognitively healthy controls, AD patients had decreased saliva t‐tau levels (≤300 pg/mg protein), while pTau181 levels increased significantly (≥18 pg/mg protein) in MCI. Using a cut‐off of ≥18 pg/mg protein of pTau181 for MCI and ≤300 pg/mg protein t‐tau for AD, a diagnostic accuracy of 71.4% was achieved for discriminating MCI and AD versus controls using a blinded approach.^27^ Similarly, higher levels of saliva pTau181 were additionally reported in AD patients.^57^ The varying results may be attributable to the methods of sample collection and specific assays employed for biomarker testing, and the authors discouraged the use of devices such as Salivette for AD biomarker analysis due to the binding of the analytes under investigation to the cotton material used in this particular device.^27^

Of note, however, the ratios of specific pTau residues to t‐tau, that is, the pTau396/t‐tau ratios for the S396 phosphorylation site are markedly elevated in the saliva of individuals with AD, suggesting the diagnostic potential of these biomarker ratios.^42^ However, the authors found no significant correlation of these biomarker ratios with either CSF tau or brain measures, for example, hippocampal volume. It is important to note that the authors observed a significant variation in the AD salivary tau levels and relatively low sensitivity and specificity of pTau396/t‐tau levels to distinguish AD and normal controls and, thus, cautioned against the utility of this test as a clinical biomarker. This variability may be attributed to variations in pre‐analytical variables and sampling, as there seemed to be inconsistent timing in collection, which may reflect potential diurnal variations, “although,” the authors noted, “collection was carried out in the morning whenever possible to control for possible diurnal variation of salivary tau.”

#### 2.1.4 Salivary NfL

NfL, a key neurodegenerative marker associated with neuronal damage, specifically axonal injury, has also been explored in saliva. Most recently, studies have explored the feasibility of measuring NfL in saliva as a potential biomarker for AD‐related neurodegeneration. Results showed that, though it is detectable and directly associated with traumatic brain injuries in college students,^63^ limitations in the diagnostic potential for AD exist.^53^ Specifically, using the Single Molecule Array (SIMOA) technology (Quanterix, USA), the authors detected no statistically significant differences in salivary NfL concentrations across the diagnostic groups comprising healthy controls, MCI, AD, and non‐AD. In contrast, they found significant plasma NfL increases in dementia cases. They also found no association between salivary NfL and other outcomes, including plasma NfL levels, CSF Aβ42, pTau181, or tau concentrations, suggesting that NfL concentration in saliva does not reflect neurodegeneration but underscores the need for improved and standardized saliva collection and sample handling for optimal biofluid analysis and developing reliable NfL saliva assays.

#### 2.1.5 Salivary GFAP

Though still in its infancy, research into salivary GFAP, an astrocytic biomarker associated with neuroinflammation in AD, is promising, correlating with the increasing roles of glial cells and microglia in AD pathophysiology.^64^ Salivary GFAP was significantly reduced in patients with MCI and AD, establishing it as a potential biomarker for distinguishing controls from those with MCI or AD. GFAP levels significantly correlated with AD biomarkers, including Aβ42, IL‐1β, and caspase‐8.^30^ Further studies are needed to evaluate the actual diagnostic and prognostic value of this biomarker.

#### 2.1.6 Acetylcholinesterase activity

Three studies investigated the activity of AChE in saliva, with conflicting results. A study where saliva samples were immediately placed on ice and subsequently stored at −70°C degrees after centrifugation showed increased AChE activity,^39^ whereas two other studies showed either a decrease in activity or no differences between the groups when samples were stored at −20°C degrees.^38^, ^41^

### 2.2 Salivary biomarkers of non‐canonical AD hallmarks and pathologies

#### 2.2.1 Overview

The significance of steroid hormone biomarkers lies in the potential for non‐invasive and easily accessible detection of hormonal, metabolic, and endocrinological changes associated with AD, providing valuable insights into the pathophysiology and progression of the disease.

#### 2.2.2 Salivary steroid hormones

Recent studies by members of our working group and others have described significant associations between alterations in salivary steroid hormones and the development or progression of AD, including preclinical disease stages.^65^, ^66^, ^67^ Cortisol, a stress hormone, and dehydroepiandrosterone (DHEA), a precursor to several sex hormones, have been mainly investigated in this context.^68^ Dysregulations in these hormone levels in saliva have been associated with cognitive decline, neurodegeneration, AD‐related pathology (reviewed elsewhere^69^, ^70^), as well as AD‐related behavioral and psychological symptoms of dementia and their improvement after non‐pharmacological treatment.^71^ Extensive research is also available on the roles of salivary sex steroids, including estrogen and testosterone, in AD (see Vest and Pike^72^). Estrogen (E2) in particular has neuroprotective effects, and changes in E2 levels can contribute to an increased risk of AD, particularly in postmenopausal women. Testosterone, though traditionally associated with male reproductive health, also plays a role in brain function and may be relevant to AD pathophysiology. For instance, the protective effects of E2 on AD‐related neuropathology have been described.^73^ While the authors did not directly measure salivary hormones, in an exciting study by Coughlan et al.,^74^ earlier age at menopause and delayed E2 hormone therapy initiation were linked to increased brain vulnerability to AD pathology.^74^ These observations suggest that specific subgroups of females, particularly during periods of reproductive health decline, may face a greater risk of pathological burden. Other studies on salivary testosterone have reported lower levels of this biomarker in individuals with incident cognitive impairment^75^ and AD.^76^, ^77^, ^78^ These studies suggest that alterations in these hormones may be linked to cognitive decline and the development of AD‐related pathology.

Despite this, challenges exist in standardizing and validating salivary hormone assays for widespread clinical use. Factors such as circadian rhythms, individual variations, and the influence of medications must be carefully considered to ensure the reliability of biomarker measurements. Further research is needed to establish robust correlations between salivary hormone levels and specific stages of AD, enhancing the clinical utility of these biomarkers.

#### 2.2.3 Salivary lactoferrin

Saliva lactoferrin, a marker indicative of the innate immune response, has recently surfaced as a potential diagnostic biomarker for AD (reviewed in a study^79^), albeit with some inconsistent findings. For example, Carro et al.^44^ demonstrated reduced levels of salivary lactoferrin in individuals with MCI and AD compared to age‐matched healthy counterparts, with high diagnostic accuracy for MCI and AD,^40^, ^44^ highlighting the crosstalk between the brain and the immune system in AD. In a follow‐up study, Reseco et al. demonstrated that salivary lactoferrin detected prodromal AD and AD dementia, distinguishing them from frontotemporal dementia (FTD) with over 87% sensitivity and 91% specificity.^40^ The work by Carro evaluating levels of lactoferrin in saliva specimens for AD is the subject of an issued Patent (WO 2017/085214) from 2017. The inventor claims specific lactoferrin levels in saliva specimens are an accurate diagnostic tool for AD. Furthermore, lactoferrin is, to our knowledge, the first established salivary biomarker for AD, and it has received regulatory approval for commercialization in Colombia.

Furthermore, salivary lactoferrin has been proposed as a potentially valuable biomarker for detecting MCI and distinguishing AD from other forms of dementia, given its correlation with the presence of brain amyloid, as documented by PET imaging studies and other neurodegenerative biomarkers.^40^, ^80^ Negative findings with this biomarker were also reported in AD patients versus controls in another study. Using the SIMOA assay, Gleerup et al. reported no differences between AD patients and controls for salivary and CSF lactoferrin.^46^ However, there may have been differences between the samples studied, pre‐analytical variables, and assays employed in the Carro studies and that of Gleerup. In the Gleerup study, the lactoferrin levels in all groups, including controls, are significantly above those reported in other studies.^81^, ^82^, ^83^ These reports warrant further investigation, potentially via head‐to‐head comparison of available assays. Apart from the evidence above other mechanistic studies have linked salivary lactoferrin with the central nervous system (CNS) pathways from both a theoretical understanding of the relationship between innate immunity and the CNS and clinical evidence, further supporting this molecule's involvement in AD.^55^, ^84^, ^85^, ^86^, ^87^ In addition, an umbrella review synthesizing findings from systematic reviews, multicenter prospective studies, and articles authored by leading experts on this salivary biomarker supports the clinical and diagnostic significance of salivary lactoferrin in AD, as it reports a more than acceptable diagnostic sensitivity ranging from 87% to 100%.^88^, ^89^ In line with the aims of this paper, standardizing salivary lactoferrin measurements has been proposed by Bartolome et al.,^88^ who demonstrated that a few pre‐analytical factors could indeed affect salivary lactoferrin levels. Importantly, salivary lactoferrin demonstrated the most significant predictive value for salivary‐based AD diagnosis based on pooled area under the curve (AUC) analysis.

#### 2.2.4 Salivary microRNA‐485‐3p

A group of researchers at the Korean company Biorchestra have discovered that the microRNA (miRNA)‐485‐3p concentration in salivary exosome‐enriched extracellular vesicles (EE‐EV) is related to Aβ deposition in the brain of patients with AD.^58^ This work confirmed that miRNA‐485‐3p concentration in EE‐EV isolated from patients with AD was significantly increased compared to that in healthy controls. Furthermore, they showed that the miRNA‐485‐3p concentration in salivary EE‐EV was significantly associated with Aβ deposition in the brain and had high diagnostic accuracy for predicting Aβ‐PET positivity.

The mixed findings around salivary AD biomarkers indicate a pressing need for further work on the validation and standardization of methods, including analytical assays, collection protocols, time of collection, and stabilization issues. Indeed, several pre‐ and post‐analytical considerations have been identified that influence the conflicting results reported, described below.

Taken together, these findings suggest that among the candidates, salivary Aβ42, tau, pTau181, and lactoferrin may be reliable markers detectable in saliva and support early AD diagnosis, with further investigations needed.

#### 2.2.5 Pre‐analytical variables for protein salivary biomarkers

##### 2.2.5.1 Collection methods

In recent years, a number of new commercial sampling methods have become available. What is apparent is that in the literature, reports vary, with missing data on pre‐analytical variables before, during, and after collection, as detailed in Table 1. In most studies, unstimulated saliva samples were obtained by spitting or drooling approximately 1 to 4 or 5 mL of whole saliva directly into a sterile polycarbonate or polypropylene tube. In a few studies,^62^, ^63^, ^64^ these tubes were precoated with a 2% sodium azide solution.^21^, ^90^ Additionally, in some studies, the Salivette collection kit (Sarstedt, Germany)^91^, ^92^, ^93^ or alternate saliva collection kits, including the SimplOFy, Super•SAL, and RNAPro•SAL kits (Oasis Diagnostics, USA),^80^ have been used for saliva sample collection. These typically can provide a more uniform or standardized specimen. It should be noted that none of the studies provided information regarding temperature and flow rate during the collection process.

##### 2.2.5.2 Post‐collection protocols

Among seven identified studies in Table 1, saliva specimens were treated with a protease inhibitor cocktail before storage.^30^, ^40^, ^42^, ^44^, ^50^, ^55^, ^58^ Note that if mass spectrometry (MS) is planned for saliva proteomics, it is crucial to carefully evaluate the use of protease inhibitors. While they can help prevent protein degradation, protease inhibitors may interfere with the later addition of proteases needed to generate peptide fragments, potentially affecting the MS analysis. In such studies, it is important to minimize protease activity by placing samples on ice immediately and freezing them as soon as possible. Only one study reported conducting a visual inspection of the sample for contaminants.^45^ After saliva collection, samples were typically placed on ice, although in a couple of studies, they were either stored at room temperature or the storage method was not described. In nearly all studies,^27^, ^28^, ^30^, ^38^, ^40^, ^42^, ^43^, ^44^, ^45^, ^46^, ^47^, ^48^, ^49^, ^50^, ^51^, ^52^, ^53^, ^54^, ^57^ saliva samples underwent centrifugation before storage at −80°C.

##### 2.2.5.3 Assay type

A plethora of different biomarkers have been explored in saliva, and an overview of the assays used to measure these biomarkers, along with their results, can be found in Table 1. Studies focusing on Aβ42 primarily employed ELISA to detect the biomarker in saliva,^29^, ^36^, ^37^, ^51^, ^52^ with other studies utilizing an immunoassay with nanobeads.^28^ The findings on salivary Aβ42 varied across studies; however, the statistically significant studies reported elevated levels of Aβ42 in patients with AD^28^, ^29^, ^36^, ^37^, ^52^ relative to those in cognitively healthy individuals. Studies investigating salivary pTau181 and t‐tau employed ELISA, Western blot, or SIMOA (Quanterix, USA), but only those utilizing ELISA or Western blot assays noted statistically significant results, specifically increased pTau181/t‐tau ratio or pTau396/t‐tau ratio in patients with AD.^26^, ^42^, ^50^, ^57^ AChE activity was measured using Ellman's colorimetric method,^38^, ^39^, ^41^ yet the results regarding AChE activity were divergent. Additionally, several studies examined salivary lactoferrin, each employing a similar ELISA kit; however, as mentioned earlier, both positive and negative results have been reported.^40^, ^44^, ^46^ Regarding NfL, only one study was identified, which did not yield statistically significant results using the SIMOA technology.^53^ In addition, one study measured salivary GFAP, with higher levels detected in patients with AD.^30^

Other studies utilized proton nuclear magnetic resonance (NMR) spectroscopy, fast ultra‐performance liquid chromatography‐MS (FUPLC‐MS), liquid chromatography‐MS (LC‐MS), ultra‐performance liquid chromatography‐tandem MS (UPLC‐MS/MS), and extended gate ion‐sensitive field‐effect transistor biosensor (EG‐IDFET) to detect new biomarkers and metabolites for AD, which are described below.^43^, ^45^, ^47^, ^48^, ^50^, ^56^

#### 2.2.6 Omics approaches for the evaluation of saliva biomarkers in AD

##### 2.2.6.1 Multi‐omics of saliva‐overview

Genomics, proteomics, and metabolomics offer powerful approaches to understanding and addressing AD, each providing unique insights into the disease's onset, progression, and potential therapeutic targets. Here, we provide examples of some advantages of specific metabolomics approaches in AD research, focusing on saliva omics markers.

##### 2.2.6.2 Metabolomics

Metabolomics examines the full array of endogenous and exogenous metabolites in biological samples. It offers a promising strategy for detecting changes in various biochemical pathways linked to the initiation and progression of AD. These metabolites, which are the end products of genomic, transcriptomic, and proteomic activities and are influenced by various external factors, including the environment, lifestyle, diet, and medications, offer a comprehensive snapshot of an organism's biochemical environment^94^ and some exciting hints on factors that may be associated to AD pathogenesis and the interplay between genetic and environmental examples.^95^

Metabolomic profiling of saliva from patients with AD has primarily been performed by mass spectrometry (MS)‐based methods, with success.^54^, ^96^, ^97^, ^98^ Using capillary electrophoresis Time‐of‐flight (TOF)‐MS, two metabolites in saliva (arginine and tyrosine) significantly differed between dementias (*n* = 10, including AD, frontotemporal lobe dementia, and Lewy body dementia) and controls.^96^ Another method utilized the process of differential chemical isotope labeling coupled with LC‐MS, specifically employing dansylation derivation, for comprehensive profiling of the amine/phenol submetabolome.^47^ In the discovery phase, 6230 metabolites were identified in saliva. Through pairwise analysis, the authors confirmed biomarkers distinguishing AD from controls (63 biomarkers), AD from MCI (47 biomarkers), and MCI from controls (two biomarkers). A panel of three metabolites effectively differentiated AD from controls and MCI, achieving a perfect AUC score of 1.0. Moreover, with positively confirmed metabolites, they could distinguish AD from controls and MCI with high diagnostic accuracy (AUC > 0.8).^47^

Marksteiner et al.^99^ utilized targeted metabolomics to investigate changes in salivary metabolites among individuals with AD and MCI and the cognitively normal, with each group comprising 25 participants. The findings revealed reduced salivary acyl‐alkyl‐phosphatidylcholine (PCae) concentrations in both the AD and MCI cohorts compared to the control group; however, significant differences were observed only in PCae C34:1‐2, PCae C36:1‐2‐3, PCaeCC38:1‐3, and PCae C40:2‐3 levels when comparing AD patients with healthy individuals. Notably, combining all these compounds enhanced the significance of the findings. Furthermore, decreased salivary levels of PCae C36:1‐2‐3 effectively distinguished MCI from healthy controls.

Using untargeted gas chromatography (GC)‐MS, the metabolomic profiles of saliva samples obtained from individuals classified as being cognitively normal or having MCI and AD (age‐ and gender‐matched, *n* = 80) were examined,^54^ collected using the RNAPro•SAL kits (Oasis Diagnostics, Vancouver, WA, USA). The metabolomics analysis yielded 173 shared metabolites across the three saliva sample groups. A pathway analysis approach revealed significant changes in multiple cellular pathways, indicating that disease progression affects several metabolic processes, including glycolysis, tyrosine, and glutathione metabolism. Partial least‐squares discriminant analysis incorporating these markers could effectively differentiate the three groups.

Another method for conducting both broad‐spectrum and specific analyses of the saliva metabolome, utilizing NMR spectroscopy alongside multivariate data analysis techniques, was used.^43^, ^45^, ^100^, ^101^ Conducting a pilot study with 12 controls, eight MCI, and nine AD patients, Yilmaz et al. were able to accurately identify significant concentration changes in 22 metabolites in the saliva of MCI and AD patients compared to controls.^43^ Additionally, statistically significant multivariate models were developed, distinguishing AD patients from control subjects and further identifying seven distinct metabolites as discriminators: acetic acid, histamine, propionate, dimethyl sulfone, glycerol, succinate, and taurine.^100^ Readers interested in a more detailed review of salivary metabolomic studies may refer to the article published by Vignoli et al., which contains a thorough review of metabolomics in AD that covers a broad spectrum of tissue types, including serum/plasma, CSF, urine, tissue extracts, and saliva.^101^

The metabolomics and proteomics findings presented here suggest that integrating multi‐omics parameters could facilitate the discovery of novel biomarkers for AD. Saliva samples hold great promise for conducting comprehensive and specific searches for AD biomarkers and understanding mechanisms. Broad‐spectrum (untargeted) and specific (targeted) metabolomic analyses can distinguish groups with AD and/or MCI from cognitively normal controls by identifying several key metabolites. The extant literature has thus demonstrated the utility and comparability of both analytical methods; however, the targeted method might be more favorable in future studies despite the fact that it is more time consuming since the filtration step that is part of this process effectively removes proteins, lipids, and other substances that could disrupt metabolite quantification and skew the results. Moreover, having a predefined list of accurately quantified metabolites will be particularly advantageous in a clinical context, while the untargeted approach will be more appropriate upstream in the research and development context.

Though novel and potentially useful in providing detailed biological insights, it is important to approach the interpretation of the biological relevance of each of the specific metabolites with some level of caution. This is because the measurements of the metabolites in whole saliva, unlike CSF and blood, are not directly exposed to the neurodegeneration processes occurring within the brain. However, as summarized here, many metabolites identified as discriminators in saliva are known to participate in metabolic pathways in AD and related dementias. Furthermore, saliva is an “ultrafiltrate” of blood, so what is in the blood is typically present in saliva, although it is sometimes present at much lower concentrations.

##### 2.2.6.3 Proteomics

Most proteomic investigations have been conducted in CSF or blood,^102^, ^103^, ^104^ which, due to its direct contact with brain cells, mirrors many processes related to neurological diseases. Advances in salivary proteomics have facilitated the detection of proteins in saliva.^103^ Notably, one study revealed that around 40% of the proteins currently employed in blood‐based diagnostic tests can also be found in saliva.^105^ The field of saliva proteomics is still in its nascent stages, with relatively few studies having been conducted so far.

Recently, Francois et al.^54^ collected saliva using RNAPro•SAL from 80 participants and showed that integrating metabolomic and proteomic analyses of saliva allows one to identify disruptions in cellular functions that could contribute to the pathology and clinical manifestations in MCI and AD. Their findings revealed significant changes in metabolites and proteins across various cellular pathways, indicating that disease progression affects a wide array of cellular functions. Using unstimulated whole saliva, changes in S100A8 and S100A9 were observed.^56^, ^106^ A cystatin interactome study demonstrated that salivary cystatin B engaged in protein–protein interactions involving numerous proteins that play crucial roles in specific biological functions such as granulocyte degranulation, neutrophil activation, modulation of the cytoskeleton, antimicrobial defense, and glucose metabolism.^107^ A preliminary quantitative analysis suggested that the decreased levels of triosephosphate isomerase in AD patients merit further investigation as a potential peripheral biomarker for AD in a larger sample of AD patients.^107^

Salivary biomarkers could provide insights into the pathogenesis of AD by using shotgun filter‐aided sample preparation proteomics, a faster and more convenient method compared to 2D LC‐MS/MS. Transthyretin (from whole unstimulated saliva) has been identified as a novel protein biomarker candidate.^108^, ^109^ Transthyretin has been reported to play roles in Aβ clearance, neuronal cell death, and gene regulation.^108^, ^110^ The reduction of transthyretin levels observed in AD subjects through LC‐MS/MS was further validated in MCI and AD subjects using Western blot analysis. Although transthyretin can be sourced from the brain and detected in saliva, 288 proteins were identified as being shared between CSF and saliva.^109^ Furthermore, transthyretin was found to regulate 14‐3‐3 proteins, a family of highly conserved acidic proteins expressed in the brain.^111^ Indeed, another study demonstrated that 14‐3‐3ȩ (stratifin) was significantly reduced in the AD group compared with controls, with simultaneous significant changes in cystatin C, haptoglobin, matrix metalloproteinase 9, and IL‐1 receptor antagonist as measured by mass spec^54^ and confirmed by ELISA.^112^

## 3 CONSIDERATIONS AND PROPOSED STANDARDIZED PROTOCOL FOR SALIVA SAMPLING, STORAGE, AND ANALYSIS

Collection, purification, stabilization, and storage of any biofluid are important factors for downstream biomarker analyses. Each sample matrix has its challenges and shortcomings, and saliva is not unique in this respect. Saliva is a highly complex aqueous biofluid containing 99% water and a multitude of components, including sodium, potassium, calcium, magnesium, bicarbonate, phosphates, immunoglobulins, proteins, enzymes, mucins, nitrogenous products, electrolytes, mucus, white blood cells, antimicrobial agents (such as urea and ammonia, secretory IgA, and lysozymes^113^), epithelial cells (from which DNA can be extracted), and salivary enzymes (including lipase and amylase).

It is well established that levels of salivary analytes are subject to pre‐analytical variables in other fields. Given the scarcity of studies examining salivary AD biomarkers that considered or reported these pre‐analytical variables, in the next section, we will critically appraise these variables in the broader context of generic salivary biomarker protocols. To gain uniformity in global cohorts, different laboratories worldwide must use the same standardized protocols in collecting saliva, regardless of the method of collection used. A consensus on how saliva collection should be performed more systematically and standardized is required, but it is not yet present in the AD field. The following section aims to achieve this goal.

Here, we break down pre‐analytical variables into four categories: (a) subject‐specific variables, (b) sample collection and processing‐related variables, (c) post‐collection sample processing, and (d) post‐processing and storage.

**a. Subject‐specific variables**

### 3.1 Salivary flow rate (SFR)

Variables that are part of donor characteristics, such as age and sex, are fixed demographic variables beyond the control of sampling researchers but can be controlled for in statistical analyses. On the other hand, other subject‐specific variables, such as consumption of medications, food and drink, and smoking, can be standardized (Table 2).

**TABLE 2:** Pre‐analytical variables pertinent to salivary AD biomarker examinations.

Phases of sampling	Controllable variables	Potentially controllable variables	Subject‐specific variables
**Before collection**	Time of collection (diurnal variation)	Dental works/oral hygiene issues	Demographic variables (eg, age, sex, ethnicity, education level)
	Fasting status	Nicotine‐containing product consumption (eg, Smoking status, vaping)	*PSEN1*, *2*, *APP*, *APOE* 𝜀4 alleles (and other AD susceptibility genes)
	Meal and drink consumption (especially foods with high sugar or caffeine content or high acidity)	Alcohol consumption	Medications
	Brushing of teeth and rinsing of mouth prior to sample collection	Diet	Gestation
	Presence of physical or psychological stressors	Activity level	Non‐AD comorbidities
		Stress level	Oral/gum diseases
		Acute infection(s) and use of complementary/alternative medicine	
**During collection**	Stimulated/unstimulated saliva		
	Location of saliva derivation/production and, thus, collection		
	Collection method (passive drool, active spitting, saliva swab or other) and tube types		
	Salivary flow rate noted		
	Temperature		
**After collection**	Addition of protease inhibitor		
	Time from collection to processing		
	Inspection of blood or other contaminants (eg, lipstick, coffee stain) visually or using laboratory tests		
	Temperature and length of time samples remain in collection tube		
	Centrifugation parameters and matrix effects		
**Post‐processing of samples and storage**	Addition of protease inhibitor		
	Time from collection to freezing		
	Temperature of freeze		
	Number of freeze‐thaw cycles		
	Volume of aliquots		

Salivary flow rate (SFR) declines with age,^114^ so age differences between patients and controls should be avoided. Pharmacological agents, including hypnotics, sedatives, antihistamines, analgesics, antipyretics, opioids, antibiotics, and vaccines, all affect salivary analytes.^115^ Notably, a common side effect of AChE inhibitors for symptomatic treatment of AD is increased saliva production. Conversely, many antidepressant drugs can cause decreased saliva flow or dry mouth (xerostomia) in subjects. Xerostomia, characterized by a reduced SFR, that is, SFR <0.1 mL/min, can be caused by several disorders, including salivary gland hypofunction^116^ and rheumatoid arthritis.^117^ Conversely, dysphagia, which is common in patients with neurological disorders associated with dementia (eg, Parkinson disease, post‐stroke period) may increase the amount of saliva in the mouth due to reduced clearance. The time taken for saliva collection should be recorded to allow the calculation of SFR, which is calculated using the formula “total salivary volume collected ÷ time taken to collect saliva (mL/min),” which in turn is used to estimate the secretion rate of the salivary analyte (concentration of analyte × SFR [µg/min]).^8^ To our knowledge, no SFR reporting has been performed in salivary AD biomarker studies. We suggest future investigators always record the SFR and keep this information together with the total volume of saliva collected. If and when the salivary biomarker of interest is known to be affected by flow rate, detailed instructions, as reported by Navazesh et al., should be followed.^118^

### 3.2 Biological sex

Several studies have assessed the effect of biological sex on salivary composition and flow rate^118^, ^119^, ^120^ and found that men have a higher SFR than women.^119^, ^120^ Furthermore, Li‐Hui et al.^121^ found lower saliva pH levels in women. Total protein concentration and saliva composition also differ between sexes.

### 3.3 Other variables affecting SFR

IL‐6 levels change with flow rate.^122^ Hence, if the flow rate is not noted during collection, the levels of salivary biomarkers may be inaccurate. The flow rate can also be affected by the viscosity of saliva, as flow rate and viscosity of saliva are positively correlated in both stimulated and unstimulated saliva.^123^ In a separate study, Mohamed et al. showed that the flow rate for different collection methods (with or without stimulation and with different types of stimulants) were as follows^20^: Flow rates for resting collection were the lowest at 0.52 ± 0.22 mL/min, mechanically stimulated saliva was the highest at 1.41 ± 0.61 mL/min, and acid‐stimulated saliva was in between, with a flow rate of 0.79 ± 0.34 mL/min.

Saliva plays a significant role in the maintenance of a balanced oral homeostasis. When salivary flow is reduced, oral infections and tooth decay can develop. Oral infections, gum disease, and reduced salivary flow can all lead to blood in the saliva, also known as hemoptysis. Since a decline in oral health is common in patients with dementia,^124^ it is important to ascertain if any blood contamination from bleeding gums is an issue since this could interfere with quantifying specific salivary biomarkers. Since a robust link was recently established between periodontitis and the risk of AD,^125^ blood interference and periodontal status have become more important.

### 3.4 Food, drink, and nicotine product consumption

The consumption of food and drink, as well as nicotine products, can affect saliva composition and flow rate. For blood‐based AD biomarkers, acute food intake alters plasma AD biomarkers in obese, but otherwise healthy, adults,^126^ particularly affecting GFAP and pTau181 the most. Most devices and manufacturers of technologies for saliva specimen collection recommend refraining from eating, drinking, smoking, and brushing teeth for a predetermined time before sample collection. Consumption of foods affects salivary biomarkers by (1) increasing the secretion of digestive enzymes that might interact with the biomarkers of interest and (2) matrix effects caused by food components and residues. Strahler et al.^115^ and Adibi et al.^127^ found that acidic food stimulates saliva production/secretions even more than sugar‐ or carbohydrate‐rich food.^128^ Saliva includes mucinous materials, and the viscosity of mucins is known to be the greatest at pH 4. Low pH and high acidity can induce proteolysis, and one of the effects of this is a collapse in the gel structure of mucins. High acidity may also cause conformational changes in salivary proteins; however, changing the pH of saliva has the advantage of inducing precipitation of mucins and decreased the viscosity of saliva in general, thereby minimizing any potential pipetting errors.^99^, ^123^

Nicotine found in cigarettes can also interfere with salivary biomarker quantification,^129^ so subjects should be instructed to refrain from smoking/vaping/consuming nicotine‐containing products in the 4 h before sampling and should not have drunk any alcohol in the preceding 12 h.^130^ Those collecting saliva specimens are advised to note smoking or alcohol consumption if these instructions are not followed.

### 3.5 Dental work

Before sample collection, no teeth brushing or use of any oral hygiene products 1–2 h prior to collection to minimize blood contamination that would likely lead to falsely elevated biomarker levels. Lastly, dental alloy restorations can release lead and cadmium into bodily fluids, and they can create a confounding factor in cases where salivary levels of these metals are to be examined.^19^ Hence, saliva sampling close in time to dental work should be avoided.

### 3.6 Physical and mental stressors, including sleep

Physical and mental stressors, including cognitive stressors and sleep, can also impact salivary biomarker levels. Physical activity increases salivary cortisol and chromogranin A (CgA) levels.^131^, ^132^ Exercise also increases both the concentration and secretion rate of lactoferrin but does not affect the secretion rate of secretory immunoglobulin A (sIgA).^133^, ^134^ Acute exercise has been shown to influence other blood‐based biomarkers related to neurodegeneration.^135^ For these reasons, participants should abstain from strenuous physical activity before saliva sampling. If needed, the exercise must be at least 24 h, ideally 48 h, before collection. Meanwhile, salivary cortisol is elevated for mental stressors with increased stress.^136^ Cognitive stressors, such as stress induced by neurocognitive assessments typically performed in AD research, similarly affect salivary cortisol and amylase levels^137^; hence, sampling should be performed before these assessments. Additionally, sleep disturbances were shown to be correlated with decreased morning awakening salivary cortisol^138^ in patients with insomnia,^138^ while no correlations were detected in healthy middle‐aged adults.^139^

### 3.7 Acute infection

Any recent acute infection, especially upper respiratory tract infections within the past 2 weeks, must be noted, as immune and related salivary markers are elevated due to infections, including COVID‐19 infections.^140^ Ideally, the subjects should be instructed to revisit and provide samples 2 weeks later.

### 3.8 Diurnal variations

Standardized single‐time‐point collections have been proposed to minimize diurnal variations for measuring salivary biomarkers in other fields. Koh et al. recommended a single measurement to be performed in the afternoon.^8^ This timing avoids the (high) cortisol awakening response; typically, biomarker levels are relatively stable by afternoon. In contrast, Henson et al. advocated collection timing of 8 to 10 a.m.^15^ Similar to salivary cortisol, salivary IL‐1β and IL‐6 levels fluctuate throughout the day.^141^ Serial samplings across 24/48 h are advised for biomarkers affected by diurnal variation, and statistical analyses using the area under the concentration versus time curve for zero are preferred for more accurate measurements.^142^

**b. Sample collection and processing**

### 3.9 Collection methods

#### 3.9.1 Collection materials

Various saliva collection methods fall broadly into two categories: non‐absorbent‐pad‐based and absorbent‐pad‐based methods. The non‐absorbent‐pad‐based methods include (1) direct spitting, that is, expectoration, of an approximate volume of saliva into a tube, (2) passive drooling of saliva through a funnel or straw into a tube, and (3) oral rinsing (swishing and gargling with 5 mL of food‐grade citric acid [0.25%] or saline solution in the mouth for 15 s, then expectorating into a tube^20^). In absorbent, pad‐based methods, saliva is absorbed passively by the presence of an inert absorbent pad or a cotton roll placed in the mouth and allowed to “wick‐up” saliva by capillary action or, in some cases, collected by chewing on the pad material, sucking, and rubbing along the gum lines or under the tongue where saliva pools. Saliva is then separated by compression of the absorbent pad or removal of the saliva from the devices by vortexing or centrifugation. In certain cases, the absorbent material acts to remove interfering substances, for example, mucins and other inhibiting factors from the specimen, providing a cleaner specimen for downstream testing; however, in the case of AD, it has been shown that Aβ species and other analytes, particularly hormones, are retained by collection kits made specifically of cotton materials, thereby impeding their correct quantification,^27^ so cotton materials should be avoided. On the other hand, materials consisting of inert polypropylene or polyethylene materials do not suffer from these issues.

#### 3.9.2 Means of collection and location of saliva derivation

Passive drooling is the most commonly used method and a number of different drool technologies are available, as mentioned earlier; however, absorbent‐pad‐based methods are gaining acceptance and application due to their relative ease of use, especially with older individuals, adults who dislike expectorating (spitting), and those with cognitive issues. Passive drooling provides whole, “mixed,” resting‐state saliva comprising saliva from all of the major salivary glands. As shown in Table 3, many technologies are available to obtain results equivalent to passive drool in the form of saliva collection kits from commercial manufacturers. Examples of devices available include Salivette (Sarstedt, Germany), Oragene and OraCollect (DNA Genotek, Ottawa, Canada), and Super•SAL, Pure•SAL, SimplOFy, RNAPro•SAL, and Micro•SAL (Oasis Diagnostics, Vancouver, WA, USA).

**TABLE 3:** Selection of commercial saliva collection kits with application in ADRD research. In general laboratory testing for diseases, a plethora of technologies may now be used to collect saliva in a standardized fashion, for hormones, drugs of abuse, infectious diseases, antibody testing, and others. General agreement exists between methodologies for testing saliva specimens since saliva has been used as a specimen routinely for decades. In the ADRD field, the situation is quite different in that saliva has become a very interesting specimen type for researchers to include in new cohorts, but the history of saliva in this area of research is relatively new. As this manuscript details, the number of studies using saliva specimens in ADRD is relatively small but rapidly increasing as organizations such as the Alzheimer's Association and the Davos Alzheimer's Collaborative investigate saliva as a biofluid for biomarker discovery in AD patients. Still, it is important to understand that work is to be done before saliva becomes a mainstream testing specimen. While a number of technologies have been used for specimen collection in the ADRD field, the overwhelming evidence points to the fact that most studies have focused on whole saliva collection using a number of different commercial kits but that newer innovations using absorbent pad‐based methods are starting to offer options for unique opportunities to look at analytes such as exosomes, cell‐free DNA, miRNAs, and others in saliva specimens. Below is a selection of saliva specimen collection devices that have found application in ADRD research so far.

1) Passive drool saliva collection kits		
Oragene‐Dx (DNA Genotek)	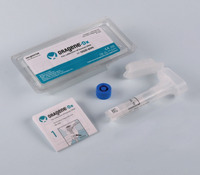	The Oragene‐Dx Device from DNA Genotek is a saliva collection kit that collects a saliva specimen by expectoration. A total of 2.0 mL of saliva is collected, which is immediately stabilized for DNA by closing the cap of the device on the collection funnel used to collect the specimen.
ORAcollect•Dx (DNA Genotek)	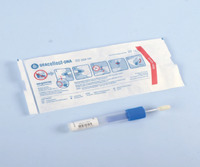	ORAcollect•Dx is designed to collect, stabilize, and transport DNA samples using a swab tip to collect specimens. Upon collection the swab is transferred to a liquid buffer to stabilize the specimen. DNA from ORAcollect•Dx can be used for research or genetic testing.
SDNA‐100 (Spectrum Solutions)	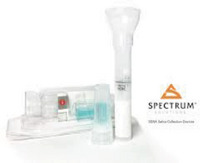	With SDNA‐100, saliva is expectorated into a funnel and patients collect until saliva reaches a black wavy line on the device signifying sufficient sample (2.0 mL). The funnel is removed and in its place a plastic vial containing stabilizing buffer is screwed onto the collection tube containing saliva. As the plastic vial is screwed down, stabilizing buffer is released into the saliva, securing the specimen for downstream applications.
SimplOFy (Oasis Diagnostics)	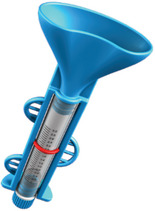	SimplOFy collects 2.0 mL saliva by expectoration. Upon collection, the tube and cap are unscrewed from the plastic housing and the cap (containing a dried‐down stabilizing buffer) is placed on top of the tube to stabilize the specimen. Two formats of the product are available for stabilization of proteins and DNA.
**2) Absorbent pad‐based saliva collection kits**		
Salivette (Sarstedt)	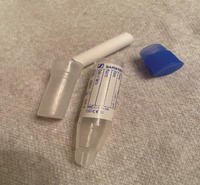	To collect saliva using Salivette, the subject chews on the absorbent pad provided for 60 s, then places the absorbent pad in the plastic tube provided. The tube is sealed using the cap provided, then the sample is centrifuged to obtain the saliva specimen for analysis. The sample is amenable to universal testing of saliva specimens.
Pure•SAL (Oasis Diagnostics)	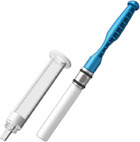	Pure•SAL collects saliva in identical fashion to Super•SAL. An additional matrix in the compression barrel of the device removes cells, providing a highly purified sample of saliva rich in proteins, cell‐free DNA, cell‐free RNA, and extracellular vesicles. Pure•SAL collects a cell‐free, highly purified specimen.
RNAPro•SAL (Oasis Diagnostics)	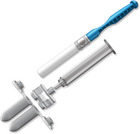	RNAPro•SAL simultaneously collects two specimens of saliva in identical fashion to both Super•SAL and Pure•SAL. Following collection, the specimen is squeezed through the provided compression tube that is connected to a bifurcation unit that splits the saliva specimen in two.

*Note*: Here is a brief summary of the pros and cons of commercial saliva collection kit types. In general, whole saliva collection kits collect 2 to 5 mL of saliva by expectoration. All molecules of interest are present in whole saliva, but to remove interfering substances, the sample must be processed. This typically requires centrifugation or some other separation method. In addition, the analyte of interest – eg, DNA, RNA, proteins, metabolites – will need to be purified to isolate the biomarker type of interest. Stabilization of whole saliva specimens is relatively easy, and samples may be stored at −80°C for long‐term storage. Whole saliva collection is generally well received, but certain older adult populations, cultures, and patients suffering from dry mouth have difficulty collecting whole saliva. Newer technologies using absorbent pads and a “passive collection” methodology eliminate the centrifuge steps needed with whole saliva by removing interfering specimens such as mucins and other interfering substances during the collection process. By modifications to these technologies, removal of cells can be carried out in addition to the removal of mucins, allowing for the immediate isolation of cell‐free DNA, cell‐free RNA, exosomes, microRNAs, and other important analytes for future ADRD research. Conversely, the drawbacks of utilizing these kits include a learning curve to accurately handle the specific steps/procedures required of the kits and the higher costs involved in purchasing the kits compared to whole saliva collection using standard wet laboratory tubes.

Singhal et al. concluded that the passive drool method was the best for AD and other neurological biomarker examinations.^21^ Recently, technologies based on non‐cotton‐based materials have emerged and should be considered and investigated.

Another variable to be aware of is the collection of “stimulated” saliva versus “unstimulated” or “resting” saliva. “Stimulated” saliva involves some stimulatory action to increase saliva flow (eg, chewing and sucking on pad‐based materials that can contain acidic compounds as an extra stimulant, chewing gum, the use of Parafilm wax, or the use of citrus fruits). Stimulation can result in artificially altered SFRs and/or localized secretion from specific salivary glands, leading to a different composition of saliva and dilution/overrepresentation of analytes of interest, potentially skewing the results. Conversely, “resting” whole saliva is mainly produced by the submandibular gland (approximately 72%), while the parotid and sublingual glands produce only 20% and 8% of whole saliva, respectively.^22^, ^23^ Regardless of the stimulation method applied, stimulated saliva is mainly derived from the parotid gland^22^, ^24^ and results in saliva composed mainly of water. This causes unwanted dilution of analytes, as confirmed by Mohamed et al., who showed that levels of smaller proteins were significantly lower in stimulated saliva samples.^20^

Furthermore, Shi et al. speculated that “stimulated” saliva might have contributed to much higher pTau181 compared to t‐tau levels than expected in their study.^26^ As a result, they urged a comparison of methods for saliva collection in future studies. Stimulation by oral rinsing has the same effect on saliva composition and analyte concentration. Direct spitting also affects secretion rate and analyte concentration due to stimulatory effects^143^ and confounding by bacteria.^144^ Chewing on gum or candy likely changes the pH of saliva, which can also interfere with downstream assays. Slowey and Cole^145^ showed non‐significant but lower levels of AD biomarkers when using the stimulated saliva method. Corroborating this, acid stimulation decreased total protein concentrations. IgE concentrations were also lowered when saliva was collected by the acid stimulation method compared to the unstimulated collection procedure.^20^

It is surprisingly common in AD and non‐AD studies to employ direct spitting, which is to be avoided, partly because it more often leads to the collection of stimulated saliva than passive drooling.^143^ This issue is particularly pertinent based on our field experience, which indicates that instead of whole saliva, subjects tend to cough up phlegm and mucus when being asked to expectorate, whether partly owing to difficulties in understanding and executing the task of spitting^143^ due to language deficits or apraxia, characteristics of MCI and AD,^146^ or owing to comorbid chronic obstructive pulmonary disease.^147^ Finally, when using non‐absorbent‐pad‐based methods, recording the time and duration of saliva collection to calculate the SFR is critical. This is not necessary with absorbent‐pad‐based methods but may be recorded if suggested by the specific manufacturer/researcher.

### 3.10 Examples of commercial saliva collection kits with applications in AD research

In dentistry, there are specialized collection devices for collecting saliva samples from specific salivary glands. For example, the modified Lashley cup or Carlson‐Crittenden device is used to collect saliva from the parotid gland, while the Wharton duct and Wolff saliva collection method are used to collect saliva from the submandibular and sublingual glands.^118^ Most studies have used the passive drool method for salivary AD biomarkers, which collects whole saliva. For most purposes, a pool of whole saliva derived from all three major salivary glands is recommended, as the three major salivary glands secrete different analytes at different rates. Typically, saliva collection kits, provided by commercial manufacturers, collect whole saliva as derived from all of the major salivary glands.

Currently, a number of manufacturers provide saliva collection kits that may be important in future AD research for salivary biomarkers. A selection of technologies currently finding application in this area is presented in Table 3.

### 3.11 Collection materials

The “cotton interference effect” also makes salivary Aβ40, Aβ42, IGF‐I, and IGF‐II undetectable in saliva samples extracted from cotton rolls. α‐amylase, IL‐1β, and TNF‐α levels were also significantly reduced compared to the passive drool collection method.^21^ Cotton swab material also causes falsely decreased IgA levels and elevated levels of hormones, including progesterone, testosterone, and estradiol.^137^, ^148^, ^149^ Li et al. also noticed that IgA and α‐amylase were lower and cortisol levels were higher using a cotton roll collection method.^91^ Reasons for discrepancies were unknown but were speculated to be due to interfering substances present in the cotton swabs^90^ or the formation of chemical bonds between salivary biomarkers in solution and the cotton fiber material.^21^

The collection tube material, that is, polyethylene versus polypropylene tubes, should also be carefully considered. For instance, low‐affinity plastic collection tubes are recommended for downstream hormone analyses to prevent the binding of salivary biomarkers to the walls of the tube.^150^ Moving forward, due to the possible adsorption of analytes to certain types of plastic,^115^ saliva samples should be collected in high‐grade polypropylene tubes or using commercially available saliva collection technologies that have been rigorously tested.

### 3.12 Self or assisted collection

Saliva collection can be performed by participants (self‐collection) or assisted/instructed by a research staff member/healthcare staff (assisted collection). In the case of participants/patients who are frail and/or have cognitive issues hampering their ability to follow instructions, research staff members/healthcare staff can assist. The main advantage of self‐collection is that patients do not need to leave their homes or do point‐of‐care testing at a remote site,^151^ while the main advantage of assisted collection is a more uniform and standardized collection procedure.

While assisted collection requires participants to travel to the research site or researchers to come to them, currently, this minor inconvenience in saliva collection is heavily outweighed by several notable advantages. For instance, a trained saliva collector can ensure the subject adheres to instructions in the pre‐analytic phase and verify the health condition of the participant; the timing and duration of saliva collection can be controlled by the trained collector, allowing measurement of flow and secretion rates; and inspection of gross adulteration of saliva (eg, by blood, food, lipstick) can also be carried out, thereby allowing rectification of any pre‐analytical variable‐related issues and allow re‐collection of the specimen on the spot. While some of these can similarly be done with self‐collection of samples by participants/patients at home, the variability/heterogeneity in pre‐analytical variables can result.

In terms of the volume required, 2 to 3 mL of total saliva specimen (passive drool) should be collected before centrifugation, which will provide an adequate amount to allow the saliva supernatant to be aliquoted into 500‐µL portions after centrifugation for storage. In the case of commercial devices, particularly absorbent‐pad‐based methods, they often do not require centrifugation, but instructions from the manufacturer should be considered.

### 3.13 Timing of sampling

Circadian rhythms govern the production, analyte composition, and flow rate of saliva.^152^ The diurnal patterns of salivary cortisol and IgA have been described elsewhere.^142^, ^153^ Diurnal variations of salivary canonical AD markers listed in Table 2 have yet to be characterized and are thus unknown, necessitating future investigation.

Before the diurnal variations and effects on salivary canonical AD markers are established, one proposal to minimize and potentially reduce any unknown effects is to sample using a standardized single time window for collection until the effects of the timing of sample collection can be verified. As an example, all sampling could be performed between 9 and 11 a.m. and the same time window used during any follow‐up testing. This may not always be possible due to logistical restrictions, but efforts should be made to standardize timing where feasible.

### 3.14 Collection and transport temperature

To ensure the stability of the saliva sample, a study proposed that participants place the collection device on ice while collecting saliva samples.^15^ Due to logistical challenges and infeasibility with older adults with cognitive issues, we suggest collecting saliva samples at room temperature, then immediately placing the collection device containing the collected samples on ice to minimize proteolytic activity by enzymes and bacteria in saliva.^123^, ^130^ It is generally known that proteins can be unstable and should be stabilized immediately after collection by adding a protease inhibitor or immediate freezing. This is true in the case of AD biomarkers; however, in the case of non‐canonical biomarkers, such as steroid hormones (eg, cortisol, testosterone), these are typically stable at ambient temperature for extended periods of time. For uncharacterized or novel biomarkers, we suggest more conservative precautions, such as collection at 4°C, adding a protease inhibitor, or flash freezing. Specifically for downstream salivary RNA examination, there is a special instruction to shake the tubes vigorously for a minimum of 8 seconds (to mix saliva with preservative solution).

**c. Post‐collection sample processing**

### 3.15 Addition of protease/RNase and/or DNase inhibitors based on type of downstream salivary biomarkers of interest (proteins, RNA, and/or DNA, respectively)

Adding the respective inhibitor immediately after specimen collection is highly recommended to preserve the integrity of biomarkers in the solution. Saliva is a biofluid rich in proteins, DNA, and RNA, so a single collection of whole saliva may measure an array of biomarkers (see Appendix 1 noting the special instructions for RNA and proteins, for example). A cocktail of inhibitors may be needed to preserve each molecular type in the saliva, and multiple aliquots of saliva may be needed to compensate for each specific application required. Typically, excess saliva collected may be used for biobanking purposes for future clinical trial protocols, future novel biomarker research and discovery, selection of patients for future therapeutic algorithms, and other applications. It is important to note that proteolytic enzymes in the saliva mainly originate from oral bacteria.^92^, ^148^, ^154^

Furthermore, saliva consists of proline‐rich proteins, immunoglobulins,^155^, ^156^ histatins, statherins,^157^ and cystatins, which are vulnerable to the actions of proteolytic enzymes, that can undergo rapid degradation. In the case of sample protection, the purposes of the addition of inhibitors are threefold: to retard bacterial enzymatic activity, protect against oral antibacterial enzymatic activity, and to prevent centrifugation‐induced release of bacterial enzymes.^156^ Notably, as Aβ42 species are noted to be sticky, McGeer and Lee and their collaborators added 0.5 mg thioflavin‐S after sample collection to prevent aggregation that could interfere with the sample assay process.

### 3.16 Inspection of blood or other contaminants (eg, lipstick, coffee), either visually or using laboratory tests

Blood contamination is the most common source of falsely elevated salivary biomarker levels. Other contaminants, including lipstick and coffee contamination, can result in falsely elevated levels of biomarkers.

As a precaution, we recommend a minimum requirement of performing a visual inspection immediately after specimen collection to examine for any gross contamination of saliva by blood or the presence of other colored materials suggesting contamination or impurities in the samples. If color staining is noted, the researcher should request another round of collections while the participants are still in attendance. The most robust method to test for contaminants, especially visually undetectable contaminants, is to perform a laboratory test. Specifically, it is helpful to test for blood contamination when salivary biomarker readings are abnormally high, possibly due to blood contamination in the saliva samples.

### 3.17 Temperature and length of time specimens stay in collection tubes (variables from collection time to temporary storage)

Storage at −20°C or lower is recommended, and in the case of passive drool, centrifugation is recommended as soon as possible (ideally within 1 h) after sample collection. In the case of absorbent‐pad‐based methodologies that provide purified saliva specimens, centrifugation may be eliminated with caution by removing interfering substances (eg, mucins) during the collection process. The two main reasons for sample storage at −20°C are to prevent bacterial growth and to ensure stability, especially protein markers, which may also be mitigated using protease inhibitors, as mentioned earlier. The best‐case scenario is to centrifuge the sample within 1 h of collection^15^ or as soon as possible^123^ where appropriate. Delayed processing of the sample (centrifugation) and storage may cause protein degradation.^123^

### 3.18 Brief vortexing, centrifugation parameters, and associated matrix effects

After finishing the collection, whole saliva should be centrifuged to separate cells and debris as soon as possible, at 4°C, to retard enzymatic activity during centrifugation. In the case of absorbent‐pad‐based methods, the specimen may be sufficiently pure without centrifugation,^158^ due to the removal of interfering factors during the collection process, but this should be evaluated on a case‐by‐case basis. For centrifugation speed, 1000 × *g* is the most commonly used speed to remove the debris and turbidity of the saliva,^123^ although some researchers recommend a higher speed to remove bacteria, cellular debris, and high molecular weight glycoproteins.^93^ Increasing the speed, however, significantly reduces total protein levels in the specimen, with a concomitant increase in larger proteins in the centrifuged samples. Increasing speed was found not to affect smaller proteins.^20^ The author reasoned that the high centrifugation force pulled larger proteins from the matrix.^20^

Mohamed et al. used a centrifugal filter device from Millipore to concentrate analytes with low molecular mass, which resulted in increased total protein concentrations.^20^ The authors suggest this could be a plausible way to concentrate analytes to enable the detection of lower‐abundance molecules. This may be pertinent for salivary AD biomarkers as studies may not detect salivary Aβ42 with low concentrations. One consideration for using this is the size of the microfilter, which should be smaller than the analytes of interest to retain the biomarker in the centrifuged samples.

Another study suggested that salivary protein determination might be affected by the rheological properties of saliva, including viscosity and gel‐forming properties, which may cause loss of biomarkers in the centrifugation process.^159^ Another study showed that patients with AD had a higher salivary total protein concentration.^112^

It is worth noting that re‐centrifugation may be required if impurities present in the specimen are significant and also due to the specific rheological properties of saliva. Repeat centrifugation could enable better clarification of the samples and removal of impurities.^15^, ^130^ Despite the work done so far, there is still a need for additional future studies to find the optimal speed of centrifugation to balance the total protein loss that will impact the limit of detection in assays. This is especially true for low‐level analytes, which can include AD biomarkers in saliva specimens.

**d. Post‐processing and storage of specimens**

### 3.19 Storage temperature and speed of freezing and thawing

After centrifugation, samples containing supernatants should be aliquoted into polypropylene cryovials to prevent repeated freeze‐thaw cycles. After aliquoting, samples should be frozen at −80°C to preserve the integrity of the analytes unless testing is to be performed within a short time. For short‐term storage (up to 3 months), specimens may be stored at −20°C. For longer‐term storage −80°C should be used. Literature reports indicate that specific metabolomic biomarkers were stable at −30°C for up to 3 months, after which protein concentrations decreased significantly at 8 months and beyond.^160^ In another MS report, storage of specimens at −20°C resulted in spectral changes, even though the samples were protected by adding a protease inhibitor cocktail.^123^ Storage at −80°C can help retain the pH of saliva while arresting the metabolic activity of bacteria that remains after centrifugation.^123^ Slow freezing, repeated freeze‐thaw cycles, and extended storage time are to be avoided for both proteins and DNA,^161^, ^162^, ^163^ and to avoid sample degradation and to preserve sample integrity, we recommend that immediately after centrifugation, saliva supernatants should be stored at −80°C or protected with the addition of an appropriate inhibitor.

### 3.20 Number of freeze‐thaw cycles

Saliva specimens are not known to be robust to multiple freeze‐thaw cycles, so we suggest that a maximum of three (preferably two) freeze‐thaw cycles should be employed. Salivary proteins are typically unstable and exhibit similar properties to blood‐based proteins, so they should be handled cautiously. Freeze‐thaw cycles break up buccal cells and bacteria, which contain most enzymes that can cause degradation; these remain in saliva supernatant. On the other hand, one advantage of the freeze‐thaw process is that it breaks down mucopolysaccharides that cause viscosity in saliva samples, which could result in pipetting errors in downstream processing.^20^

### 3.21 Volume of aliquots

To avoid repeated freeze‐thaws, aliquoting the specimen is recommended. In the literature, a volume of 330 µL^15^ has been recommended. Here, we suggest a volume of no more than 500 µL, based on the volume each cryovial can hold and the typical number of specimens required for one round of testing using the chosen assay. This can vary depending on the test format, for example, ELISA, SIMOA, and MS. The recommended aliquot quantity is between 100 and 250 µL depending on the downstream assay and technology used for biomarker detection and quantification.

### 3.22 Proposed protocol for saliva collection

Please see Appendix 1.

It is worth noting that for certain collection devices, the centrifugation steps can be skipped.

In particular, pad‐based systems (Table 3), including the Super•SAL, Pure•SAL, RNAPro•SAL, and Micro•SAL saliva collection kits that function by collecting saliva passively using an inert absorbent pad, retain a high proportion of the interfering mucinous materials in saliva on the pad while the analytes to be tested remain in the saliva filtered into the collection tube provided. This action of removing interfering substances is equivalent to centrifuging a specimen, so it acts as a highly time‐saving and convenient feature of these devices.

### 3.23 Potential context of use

Due to the heterogeneity of pre‐analytical variables, assays, and technologies used, which have resulted in mixed findings, it is still premature to specify the context of using salivary biomarkers for AD diagnosis in research and clinical settings. However, below, we offer a few potential contexts of use:

- Global health context: Compared to blood and CSF sampling and biomarkers, there are distinct logistical and other advantages to applying salivary AD biomarkers in underserved populations, including racial/ethnic minorities, rural areas, and developing/underdeveloped countries. In these contexts, the accessibility and infrastructure for collecting and transporting blood and CSF, as well as freezer facilities for blood and CSF storage for AD biomarker studies, are limited or may not be available. Additionally, cultural considerations need to be taken into account. Based on our experience working in the field in East Asia and Africa, unless advised by a physician to undergo medical testing, locals tend not to agree to provide informed consent for CSF, and higher consent rates are observed for saliva compared to blood collection. While there could be potential challenges and limitations of implementing the proposed standardized protocols across various research settings, particularly in resource‐limited environments or large‐scale population studies, there are ways to circumvent these issues. For example, though it is advised to centrifuge the samples as soon as possible after sample collections, specific absorbent‐pad‐based saliva collection kits are presented in Table 3 (and described above) that negate the need for centrifugation, as the kits can perform the purification steps similar to that of centrifuging. The availability of specific facilities for proper storage of samples could pose an issue as well. On the other hand, this is the same issue that other biofluids, including blood and CSF, suffer from. One option would be to employ saliva collection on a piece of paper, similar to dried blood spot collection, which negates the need for storage at a certain temperature, which also enables easy mailing of samples. Saliva‐based biomarker research holds significant promise for early dementia diagnosis and intervention, particularly for underserved communities, including in the majority of world settings where access to more invasive procedures, such as CSF collection or even blood sampling, is limited. Saliva offers a non‐invasive, easily collectible sample and, hence, has incredible potential to democratize access to early detection tools, facilitating timely interventions in communities with limited healthcare resources. Given the reduced requirement for complex infrastructure, saliva‐based diagnostics can enhance precision health strategies and contribute to closing the gap in dementia care and prevention in these regions.
- Research versus clinical use: As indicated in one of the subsections, in a research setting, targeted approaches looking at salivary proteomics, metabolomics, and other omics should be evaluated to identify potential candidates for upstream validation for potential clinical validation and regulatory approval in the future.
- Accelerate screening and recruitment in clinical and research settings, that is, population‐based cohort studies and large‐scale clinical trials: Increase the speed of screening into clinical trials or for referrals to physicians, particularly more racially diverse and representative groups performed outside the clinics.
- Potential clinical use: As we look to the future, where sampling and assay procedures are standardized and salivary AD biomarker results are less heterogeneous across laboratories and more robust and replicable, we envision a growing potential diagnostic and prognostic utility for salivary AD biomarkers, including both canonical and non‐canonical groups of markers, similar to oral cancer molecular staging profiles as objective prognostic indicators.^15^
- Other potential settings and contexts of use include population screening for research (including cohort studies and clinical trials) and public health screening, campaigns, and eventual point‐of‐care devices for use in healthcare clinics and home care and self‐monitoring.

## 4 CONCLUSION AND FUTURE DIRECTIONS

Saliva constitutes a highly viable sample for AD diagnosis and longitudinal monitoring due to its distinct advantages, including minimal invasiveness, simplicity of collection, and accessibility for research participants and patients, especially outside clinics. While saliva‐based biomarkers show promise for detecting AD pathology, including Aβ, tau, pTau isoforms, NfL, GFAP, and lactoferrin, their clinical utility remains unknown, warranting future investigation. Challenges, such as assay standardization, sensitivity, and specificity, must be addressed before these biomarkers can be routinely used for AD diagnosis and monitoring. Additionally, longitudinal studies are necessary to establish the reliability and predictive value of saliva‐based biomarkers in different stages of AD.

However, regardless of the potential future contexts of use, that is, in‐home testing, primary care, or trial selection, there is a need for further standardization and validation of salivary AD biomarkers, highlighting the need for further research. Future research should closely scrutinize how each variable affects AD biomarker measurements in saliva to determine the optimum conditions for collecting, stabilizing, and testing each of the new generation of biomarkers. There will likely not be a one‐size‐fits‐all pre‐analytical protocol as different salivary AD biomarkers have different physiological roles and are, therefore, affected by pre‐analytical variables differently. However, the proposed standardization protocol is the first step to achieving that. Furthermore, the availability of validated assays and technologies to collect, stabilize, and quantify protein‐based and omics analytes in saliva samples is going to be crucial. Last but not least, it is worth noting that the development of salivary biomarkers is not meant to replace but to complement other biofluids, such as CSF and blood, especially in the specific contexts of use stated earlier, that is, where it is not feasible because of the lack of infrastructure or resources and cultural acceptability. Once these factors have been ironed out, credentialing of the tests through FDA clearance and CE Mark approval will be necessary before widespread use.

## CONFLICT OF INTEREST STATEMENT

Chinedu Udeh‐Momoh has received grants, paid to her institutions, from UKRI MRC Applied Global Health Award (MR/Y019822/1), Wellcome Leap and Temasek Trust, Alzheimer's Association, Davos Alzheimer's Collaborative Global Cohort Fund, Global Brain Health Institute, UK Defence & Security Accelerator, Veterans’ Health Innovation Fund, and Rosetrees Foundation; consulting fees from Aga Khan University; support for attending meetings and/or travel from the Alzheimer's Association for symposium presentations at the LMIC and AAIC meetings; and holds unpaid leadership roles in the British Society for Neuroendocrinology, Alzheimer's Association ISTAART BBB‐PIA, National Institutes of Health (NIH)‐sponsored NASEM ADRD project, and WHO's Guideline Development Group for Cognitive Decline and Dementia Risk Reduction. Nicholas Ashton has received consulting fees from Quanterix, payment from Alamar Biosciences, Biogen, Eli Lilly, and Quanterix, holds a pending patent (PCT/US2024/037834) on Methods for Remote Blood Collection, Extraction and Analysis of Neuro Biomarkers, and has participated and received payment in advisory boards for Biogen, TargetALS, and TauRx. Henrik Zetterberg has received grants, paid to his institution, from the Swedish Research Council, European Union's Horizon Europe, Swedish State Support for Clinical Research, Alzheimer Drug Discovery Foundation, The Bluefield Project, Cure Alzheimer's Fund, the Olav Thon Foundation, the Erling‐Persson Family Foundation, Stiftelsen för Gamla Tjänarinnor, Hjärnfonden Sweden, the European Union's Horizon 2020 research and innovation programme under the Marie Skłodowska‐Curie grant agreement No 860197 (MIRIADE), the European Union Joint Programme – Neurodegenerative Disease Research (JPND2021‐00694), the National Institute for Health and Care Research University College London Hospitals Biomedical Research Centre, and the UK Dementia Research Institute at UCL (UKDRI‐1003); consulting fees from Abbvie, Acumen, Alector, Alzinova, ALZPath, Amylyx, Annexon, Apellis, Artery Therapeutics, AZTherapies, Cognito Therapeutics, CogRx, Denali, Eisai, LabCorp, Merry Life, Nervgen, Novo Nordisk, Optoceutics, Passage Bio, Pinteon Therapeutics, Prothena, Red Abbey Labs, reMYND, Roche, Samumed, Siemens Healthineers, Triplet Therapeutics, and Wave; has given lectures in symposia sponsored by Alzecure, BioArctic, Biogen, Cellectricon, Fujirebio, Lilly, Novo Nordisk, Roche, and WebMD; has participated and received payment in advisory boards for Abbvie, Acumen, Alector, Alzinova, ALZPath, Amylyx, Annexon, Apellis, Artery Therapeutics, AZTherapies, Cognito Therapeutics, CogRx, Denali, Eisai, LabCorp, Merry Life, Nervgen, Novo Nordisk, Optoceutics, Passage Bio, Pinteon Therapeutics, Prothena, Red Abbey Labs, reMYND, Roche, Samumed, Siemens Healthineers, Triplet Therapeutics, and Wave; holds unpaid leadership roles with the Alzheimer's Association Global Biomarker Standardization Consortium and chair of the IFCC WG‐BND; holds stock and is a co‐founder of Brain Biomarker Solutions in Gothenburg AB (BBS), which is a part of the GU Ventures Incubator Program (paid). Robert A. Rissman has received research support from the National Institute on Aging and the Alzheimer's Association and consulting fees from Amydis Inc., Biogen, Bioivt, Lexeo, Keystone Bio, Allyx, DiamiR, Ionis, and PrecisionMed. Charisse N. Winston has received grants U19AG074879, K99 AG070390, and R00 AG070390. Robert Jenkins is CEO of Geroa Diagnostics (paid). Kumar B. Rajan has received grants R01AG073627, R01AG058679, and UH2AG083289, paid to his institution. Anja Hviid Simonsen's institution has received funding from PharmaDanmark. Paul D. Slowey is Co‐Chair of the Saliva Working Group for Alzheimer's Disease (unpaid), Founder of North American Saliva Symposium (unpaid), Founder and CEO of Oasis Diagnostics Corporation (paid), and Chief Science Consultant for RapidDx, Inc. (stock compensation) and holds an Honorary Professorship at Central South University, China. Ted K.S. Ng, Mei‐Ann Lim, Helena Sophia Gleerup, Wayne Leifert, Catherine Ajalo, Sid O'Bryant, Eva Carro, Gorka Orive, Stefano Tamburin, Marcos Olvera‐Rojas, Patricio Solis‐Urra, Irene Esteban‐Cornejo, Gustavo Alves Andrade Dos Santos, David Koh, and Alzheimer's Association International Society to Advance Alzheimer's Research and Treatment: Biofluid Based Biomarkers Professional Interest Area Salivary Biomarkers for Dementia Research Working Group (ISTAART‐BBB‐PIA‐SWG) report no conflicts of interest. Apart from all contributing members listed on the author list, non‐contributing members of ISTAART‐BBB‐PIA‐SWG include Kimberley Mullen, Los Angeles, CA, USA; Ali Ezzati, New York, NY, USA; Chantal Bazenet, Zürich Metropolitan Area, Zürich, Switzerland; Jianhong Ching, Singapore, Singapore; Lydia LePage, San Francisco, CA, USA; Charlotte Teunissen, Amsterdam, the Netherlands. Author disclosures are available in the Supporting information.

## Supporting information



Supporting Information

## 1 Proposed protocol for saliva collection

### Preparations to start pre‐analytic phase

This protocol is detailed for the collection of human saliva, assisted collection (both the research staff and participant are present), sampling by passive drooling, saliva collection kits, biobanking (ie, long‐term storage of salivary samples), and analysis of specific analytes.

### 1.1 Guidelines to saliva collection kits

- Establish single‐time‐point collection (ie, from 9 to 11 a.m. (or another standardized time window) or several time‐point measurements in a day (if the biomarkers have diurnal variations requiring a few collections throughout the day).
- Prepare saliva kit supplies in advance prior to meeting the participant. For passive drool collection use sterile and low‐affinity polypropylene collection tubes/containers. The use of inert absorbent pad‐based methods is more than acceptable; however, the use of collection kits containing cotton material is not recommended. For saliva collection kits from commercial manufacturers, adhere strictly to the instructions for use provided by the manufacturer.

### 1.2 Before collection

Instructions to research participants 1 day before appointments for saliva collection

1. Only schedule appointments from 9 to 11 a.m. (or another standardized time window) to minimize potential diurnal variation. Re‐arrange appointments if they fall outside of this timeframe, such as scheduling conflicts or late arrivals, for example.
2. It is recommended that, due to the high turnover of saliva in the oral cavity (we produce 1.0 to 1.5 L of saliva every day), sample collection should not begin until at least 1 h after any food intake, but longer periods of time are not necessary.
3. Within this period, only consumption of plain water should be allowed. There should be no consumption of food or drinks with high sugar or caffeine content or high acidity for at least 1 h prior to collection.
4. Do not smoke in the preceding 4 h and do not drink alcohol in the preceding 12 h.
5. The effects of vaping have not yet been evaluated, so until data are available this activity should also be avoided 4 h prior to sampling.
6. Do not use any oral hygiene products, including brushing of teeth, 1 to 2 h prior to collection.
7. Do not perform vigorous physical exercise at least 24 h (ideally 48 h) until after saliva collection is completed. If needed, exercise performed must be at least 2 h prior to collection.

### 1.3 Instructions for research staff

Saliva sampling should be performed before assessments, particularly neurocognitive assessments to avoid stressors.

### 1.4 Instructions for participants during appointment

Participants need to be instructed before and during saliva collection.

### 1.5 Before saliva collection

- Register with the research staff to fill in a questionnaire and (only if resources are available at the collection sites) have the oral cavity, gums, and tongue inspected.
- Rinse the mouth with water 10 min before specimen collection.
- Rest for 10 min to relax sitting on a chair.

### 1.6 During collection: Saliva collection – passive drool

- Collect a prespecified amount of saliva, for example, 2 mL, with the varying time taken for each participant to reach the noted prespecified amount to enable saliva flow rate calculation (flow rate = saliva volume collected/time in seconds). Detailed procedural steps follow:
- Pick up the saliva collection kit provided, remove cap from vial.
- Place straw securely into vial.
- Instruct participant to:

Allow saliva to pool in the floor of the mouth for 5 min.

Rest and produce drool with minimal oral movement.

Sit in an upright position, then proceed by tilting participant's head down slightly to pool saliva in the mouth.

- Sit upright, pass the drool into the collection tube provided, and start timing with a stopwatch.
- Stop timing when the drool reaches the prespecified amount of saliva sample to be collected based on the marking made on the collection tube. Record time.
- If there is insufficient saliva, especially in the case of older adults with xerostomia or with patients on anti‐depressant drugs or others causing dry mouth, pause timing and place the vial on ice.
- Allow saliva to pool again in the floor of the mouth for 5 min.
- Continue collection and resume timing.
- Repeat, if necessary, until saliva reaches the desired volume.
- Replace cap on vial, and immediately place vial in freezer. Consider at this step whether it is necessary to add a small volume of protein stabilizing agent or protease inhibitor (eg, note these reagents must be compatible with downstream analyses such as mass spectrometry).

Specifically for downstream salivary RNA examination: Special instructions to shake tubes vigorously for a minimum of 8 s (to mix saliva with preservative solution).

*Instead of passive drool, an oral swab may be appropriate for older adults with xerostomia (dry mouth syndrome); otherwise, it may not be possible to collect the desired amount of saliva. Note on the participant's sheet if alternative methods were used.

Similarly, while saliva collection methods are non‐invasive and painless, provision of saliva by passive drooling is not necessarily convenient and feasible with respect to patients with cognitive decline, such as AD, so alternate methods available will facilitate easier sample collection in these subjects.

### 1.7 During collection: Saliva collection – commercial saliva collection kits

Follow the instructions provided by the specific device manufacturer.

### 1.8 After collection: Instructions to research staff after saliva collection

- Perform a visual inspection of the saliva sample for any coloring or other gross contaminants. If color stain is noted, the researcher should discard the original sample and request another round of collection to rectify the situation with participants on the spot.
- Transport of sample to wet laboratory at −20°C is recommended.
- For collection kits that do not purify saliva samples and thus require centrifugation, centrifugation is to be performed as soon as possible after sample collection (ideally within 1 h starting from the first collection of the day). Follow a two‐step centrifugation process to clarify the saliva prior to storage. The recommended procedure is centrifugation at 2500 × *g* at 4°C for 15 min for initial clearing, followed by centrifugation at 13,000 × *g* at 4°C for 30 min for further clarification before aliquoting the samples for storage. As mentioned earlier, this may not apply to specimens collected using commercial kits that include an absorbent pad capable of removing mucinous material from the specimen.
- Aliquot the sample according to the desired volumes into polypropylene vials.
- Send one aliquot for occult blood testing to detect potential blood contamination if gross contamination is present.
- Store pellets and supernatants separately at −20°C or −80°C, respectively. For short‐term storage (up to 3 months), supernatants may be stored at −20°C.
- Volume of aliquots: The recommended aliquot quantity is between 100 and 250 µL depending on the downstream assay and technology used for biomarker detection and quantification.
- Ensure a maximum of three (preferably two) freeze‐thaw cycles per aliquot.
